# Infection-adapted emergency hematopoiesis promotes visceral leishmaniasis

**DOI:** 10.1371/journal.ppat.1006422

**Published:** 2017-08-07

**Authors:** Belma Melda Abidin, Akil Hammami, Simona Stäger, Krista M. Heinonen

**Affiliations:** 1 INRS-Institut Armand-Frappier, Université du Québec, Laval, Québec, Canada; 2 Centre for Host-Parasite interactions, Laval, Québec, Canada; Queensland Institute of Medical Research, AUSTRALIA

## Abstract

Cells of the immune system are derived from hematopoietic stem cells (HSCs) residing in the bone marrow. HSCs become activated in response to stress, such as acute infections, which adapt the bone marrow output to the needs of the immune response. However, the impact of infection-adapted HSC activation and differentiation on the persistence of chronic infections is poorly understood. We have examined here the bone marrow outcome of chronic visceral leishmaniasis and show that the parasite *Leishmania donovani* induces HSC expansion and skews their differentiation towards non-classical myeloid progenitors with a regulatory phenotype. Our results further suggest that emergency hematopoiesis contributes to the pathogenesis of visceral leishmaniasis, as decreased HSC expansion results in a lower parasite burden. Conversely, monocytes derived in the presence of soluble factors from the infected bone marrow environment are more permissive to infection by *Leishmania*. Our results demonstrate that *L*. *donovani* is able to subvert host bone marrow emergency responses to facilitate parasite persistence, and put forward hematopoiesis as a novel therapeutic target in chronic infections.

## Author summary

Hematopoietic stem cells (HSCs) are responsible for the generation of all blood cells and thus play an important but often underappreciated role in the host response to infections. HSCs are normally dormant, but they can become activated in response to stress, such as infections. This stress response is meant to generate more blood cells and help the body to eliminate the invading pathogen. We have studied here the activation of HSCs in a mouse model of chronic infection with the parasite *Leishmania donovani*. We found that the parasite efficiently activates HSCs and steers them to produce large numbers of specific blood cells that are among the preferred targets of the parasite and become even more susceptible to infection when produced within the diseased environment. Using a mouse strain in which HSC activation cannot be sustained, we found that diminished HSC activity correlated with decreased parasite numbers. We therefore propose that HSC activation by the parasite promotes the infection and could be used as a new target for treatment.

## Introduction

Emergency hematopoiesis in response to severe bacterial infection is associated with an expansion of hematopoietic stem/progenitor cells (HSPCs) and their progeny, mediated by a combination of environmental cues, including inflammatory cytokines and microbial products [1–4]. Activated, proliferating HSPCs and myeloid progenitor cells (MPCs) leave the bone marrow and migrate to the spleen, liver, and other inflamed target organs where they can directly contribute to hematopoiesis [1, 5, 6]. In the case of acute bacterial infection, the resulting increase in myeloid differentiation promotes the immune response that is required for clearance of the pathogen [3, 4]. Similarly, increased myelopoiesis in response to an acute viral infection can be beneficial for helping to mount the appropriate T cell responses to eliminate infected cells [7]. Bone marrow homeostasis is usually restored after the infection is cleared. However, the potential impact of infection-adapted hematopoiesis on the ability of pathogens to persist and establish chronic infections has not been widely studied.

Persistent inflammatory conditions, including chronic parasitic infections such as visceral leishmaniasis may lead to an acquired bone marrow failure, where the HSPCs and bone marrow stroma are no longer able to support blood homeostasis [8–10]. The underlying mechanisms are poorly understood, but the pathology clearly suggests that the parasite infection profoundly changes normal hematopoiesis: visceral leishmaniasis is generally associated with hepatosplenomegaly, extramedullary hematopoiesis, pancytopenia, and immunosuppression. Previous studies using the Balb/c mouse model of infection with *Leishmania donovani* suggest that the parasite does not directly infect HSPCs or MPCs. Instead, *L*. *donovani* establishes a persistent infection in bone marrow macrophages, which correlates with an enhanced MPC output by bone marrow and spleen, and the production of myeloid growth factors [10–12]. However, the contribution of enhanced myelopoiesis to the course of infection is not well understood.

HSPC activation corresponds to changes in Wnt signaling activity, with the various intracellular signaling pathways promoting either activation or quiescence [13–15]. We have previously shown that the absence of the Wnt signaling receptor Frizzled6 (Fzd6) results in defective stem cell self-renewal, completely abrogating their ability to reconstitute an irradiated host after transplant, and dampens HSPC expansion during LPS-induced emergency hematopoiesis [15]. Wnt signaling generally contributes to the establishment of T cell memory and regulates effector T cell responses [16, 17] and leukocyte trafficking [18], but its role in inflammation-induced myelopoiesis and the regulation of chronic infections is not known.

We show here that experimental *L*. *donovani* infection induces the expansion of hematopoietic stem cell (HSC)-like cells and Sca1^+^ emergency MPCs in the bone marrow. The myeloid progeny of these emergency MPCs consists predominantly of Ly6C^hi^ monocytes with a regulatory, suppressor cell-like phenotype. We further demonstrate that the expansion is functionally important, as monocytes generated in the presence of soluble factors extracted from the infected bone marrow are more permissive to infection, and a stunted emergency response such as seen in *Fzd6*^-/-^ mice results in decreased parasite burden. Collectively our results support the hypothesis that *Leishmania* subverts the host bone marrow emergency response to promote its own proliferation and to allow for continued persistence of the infection.

## Results

### *L*. *donovani* induces the expansion of HSC-like cells in the bone marrow and spleen

**Fig 1:**
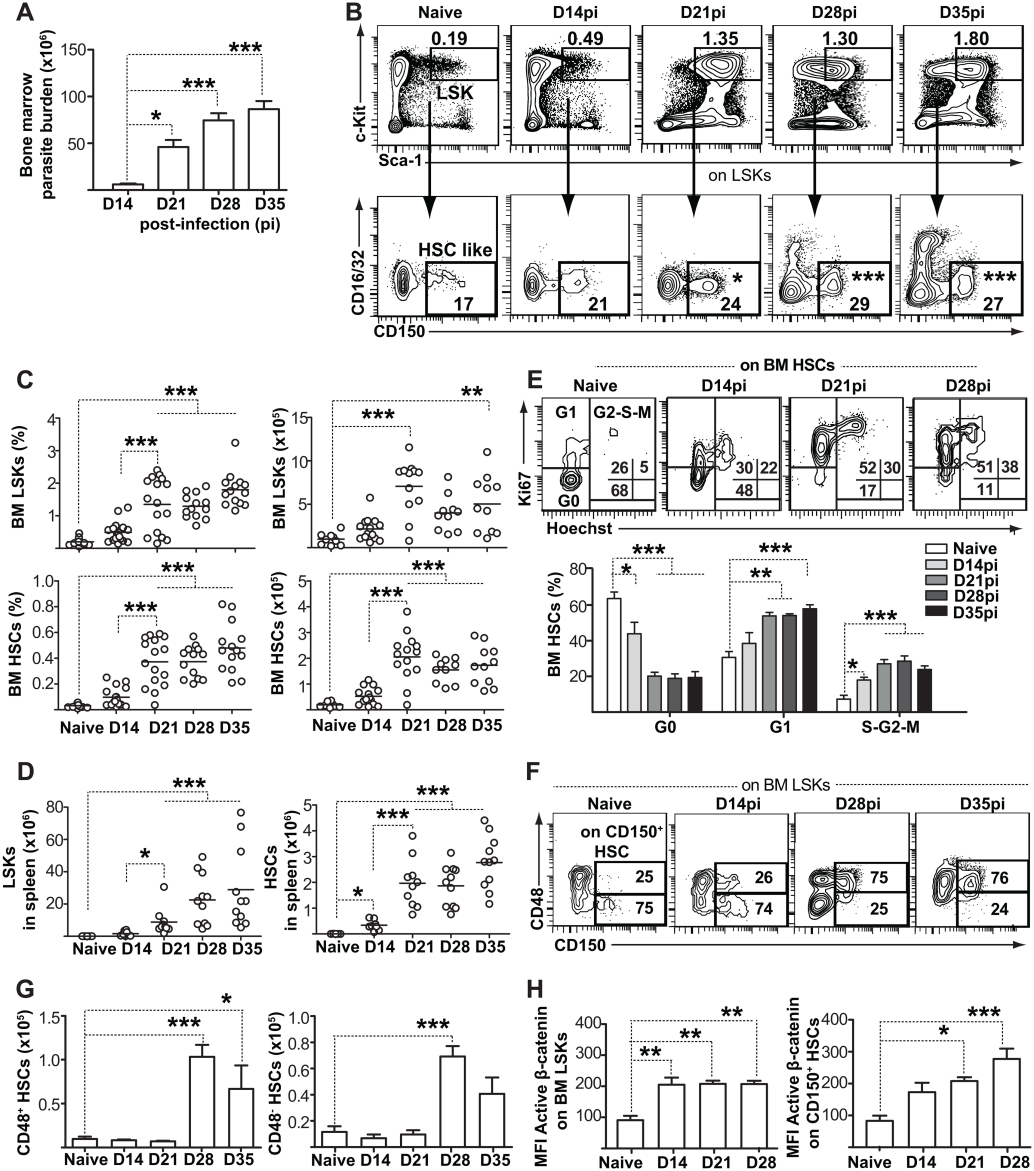
Parasite expansion coincides with proliferation and accumulation of bone marrow hematopoietic stem/progenitor cells (HSPC). **(A)** Bone marrow parasite burden was assessed using the serial limiting dilution technique. Graph shows parasite burden per one femur and one tibia. **(B)** Representative flow cytometry data and gating strategy of BM HSPCs. BM cells were first gated on Lin^-^ (B220^-^CD3ε^-^CD11b^-^GR1^-^Ter119^-^) and identified according to Sca1 and cKit (CD117) expression. HSCs were defined as CD150^+^ CD16/CD32^-^ within the Lin^-^cKit^hi^ Sca1^+^ (LSK) population. Numbers within flow cytometry plots represent mean LSK percentage within total bone marrow and mean HSC percentage within LSKs. See also S1 Fig. **(C)** Histograms show percentage and absolute numbers of LSKs and HSCs per two femora and two tibiae. Data are pooled from three independent experiments, with each individual dot representing one mouse. Horizontal lines represent the sample mean. **(D)** Absolute numbers of LSKs and CD150^+^ HSCs in spleens of infected mice. See also S1 Fig. **(E)** Ki-67/Hoechst co-staining was used to distinguish the G0, G1, and S/G2/M cell cycle phases of CD150^+^ HSC-like cells during infection. **(F)** Analysis of CD48 expression within BM CD150^+^ HSC-like cells. Numbers within flow cytometry plots represent CD48^+^ and CD48^-^ HSC subsets within CD150^+^ HSC like cells. **(G)** Graphs depict the numbers of cells for the two subsets at various time points after infection. **(H)** Intracellular active β-catenin levels (MFI) within CD150^+^ HSC-like cells. See also S1 Fig. All bar graphs represent mean + SEM with 4 mice per group coming from one single infection. Similar results were obtained in two additional independent experiments. *P<0.05; **P<0.01; ***P<0.001.

*L*. *donovani* infection results in enhanced myelopoiesis in the bone marrow and spleen of Balb/c mice [11, 12]; however, it is unclear which HSPCs the parasite targets and what functional consequences stem from the increased myeloid output. We initially followed the progression of *L*. *donovani* infection in the bone marrow of mice on C57Bl/6 background and observed a sharp increase in parasite burden beginning in the third week after infection (Fig 1A). In parallel with parasite burden, the proportion and number of bone marrow Lin^-^Sca-1^+^c-Kit^+^ (LSK) cells and CD150^+^CD16/CD32^-^LSKs, which correspond to an HSC-like phenotype in uninfected mice, also increased, reaching a plateau between day 21 and day 28, depending on the strength of the infection (Fig 1B and 1C, S1 Fig). A similar expansion was also observed in the spleen: both LSKs and HSC-like cells were virtually undetectable in the naïve spleen, but their numbers continued to augment in infected mice through day 35 (Fig 1D, S1 Fig). These results indicate that the most immature HSPCs are indeed affected by infection with *L*. *donovani*, and that this effect persists in time.

Adult HSCs are usually dormant in the bone marrow, with more than two thirds of the cells residing in a quiescent state in the G0 phase of the cell cycle. However, they become readily activated under stress or in response to inflammatory cytokines [4, 19, 20]. Chronic infection with *L*. *donovani* induced HSC-like cells to enter cell cycle, resulting in a gradual loss of quiescent cells (Fig 1E). This was accompanied by differentiation, as the proportion of CD150^+^ HSC-like cells that had acquired CD48 and thus represented multipotent progenitors with myeloid bias [19, 21] also increased from 25% to 75% (Fig 1F and 1G); however, there was a significant expansion of the CD48^-^ population as well. HSC activation and cell cycle entry have been shown to correlate with the induction of β-catenin-dependent Wnt signaling in non-infectious settings [13]. We observed a significant increase in intracellular levels of active β-catenin that was specific for CD150^+^ HSC-like cells (Fig 1H), suggesting that Wnt signaling could contribute to regulating *L*. *donovani*-induced HSC expansion.

### Induction of myelopoiesis during *L*. *donovani* infection results in the generation of altered progeny with a regulatory phenotype

**Fig 2:**
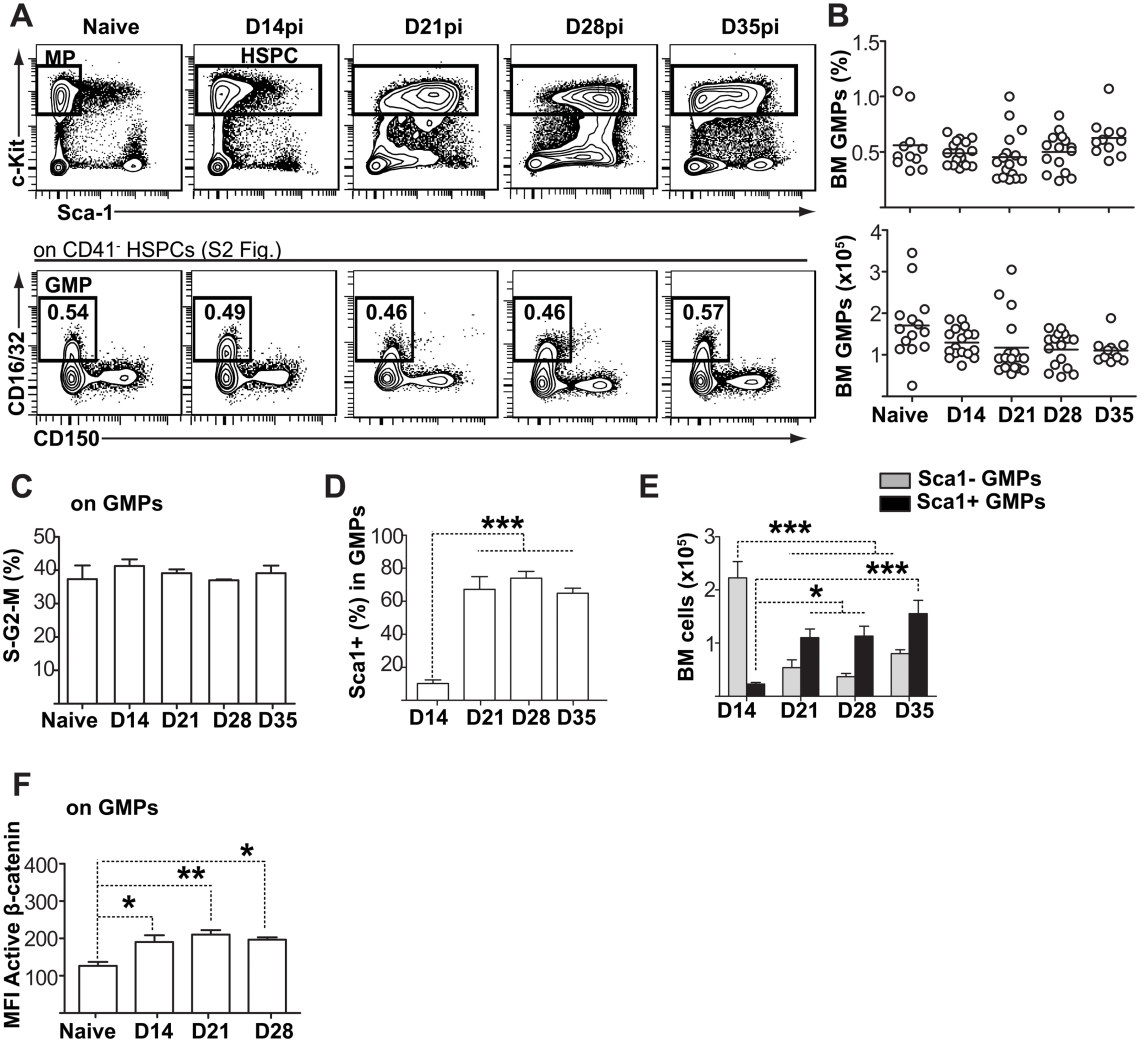
Bone marrow HSCs switch their differentiation towards non-classical myeloid progenitors. **(A)** Representative flow cytometry data and gating strategy of granulocyte-monocyte progenitors (GMPs) in the bone marrow. Steady state myeloid progenitor (MP) cells were first gated on Lin^-^Sca1^-^c-kit^hi^ and then subdivided according to the expression of CD41, CD150 and CD16/CD32. GMPs were identified as CD16/CD32^+^ CD41^-^ CD150^-^. Due to the inflammation-induced shift in Sca1 expression, the total Lin^-^ c-Kit^hi^ HSPC population was included for analysis during infection. See also S2 Fig. **(B)** Graphs show percentage and absolute numbers of GMPs at various time points. Data are pooled from three independent experiments, with each individual dot representing one mouse. Horizontal lines represent the sample mean. **(C)** Percentage of GMPs in S/G2/M phases of cell cycle. **(D)** Percentage of Sca-1^+^ emergency GMPs within all GMPs. **(E)** Numbers of cells within Sca-1^+^ and Sca-1^-^ GMP subsets. **(F)** Intracellular active β-catenin levels (MFI) in GMPs.

Leishmaniasis is accompanied by an increase in circulating monocytes, and *L*. *donovani* induces the expansion and export to spleen of MPCs in Balb/c mice [11, 22, 23]. To better define the kinetics and the types of progenitors that were responding to the infection, we analyzed the bone marrow MPC compartment and observed that the number and proportion of granulocyte-monocyte progenitors (GMPs; CD16/CD32^+^cKit^+^CD41^-^CD150^-^Lin^-^) remained stable over time (Fig 2A and 2B, S2 Fig). There was no specific change in the proportion of actively cycling GMPs (Fig 2C, S2 Fig), and in contrast to HSC-like cells, the increase in active β-catenin levels was very modest (Fig 2F). However, there was a striking upregulation of Sca1 expression on GMPs (Fig 2D and S2 Fig), which translated into a decline in the numbers of Sca1^-^GMPs that are normally associated with steady-state hematopoiesis. The steady increase in the numbers of Sca1^+^ “emergency” GMPs (Fig 2E; [24]) together with the stable levels of total GMPs (Fig 2A and 2B) suggested that the major change brought about by *L*. *donovani* did not impact as much the numbers of MPCs as the type of progeny they generated. B lymphopoiesis was nearly completely abrogated at later stages (S3 Fig), indicative of profound alterations to the bone marrow environment.

**Fig 3:**
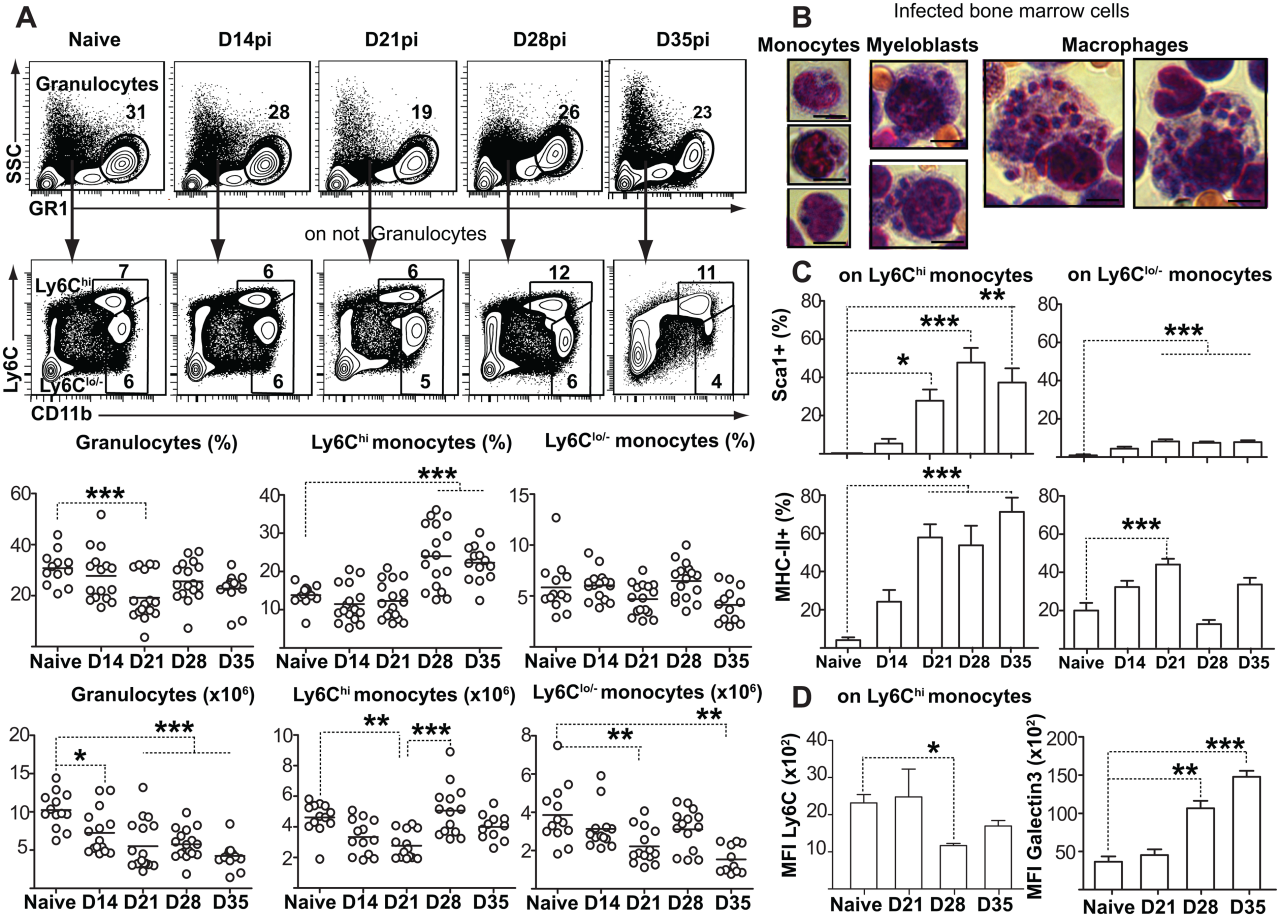
Leishmania parasite expansion promotes myeloid output in the bone marrow. **(A)** Representative flow cytometry data to demonstrate gating strategy for myeloid cell subsets in the bone marrow. Graphs show percentage and numbers of granulocytes (GR1^hi^ SSC^hi^), mature monocytes (Ly6C^hi^CD11b^+^) and remaining immature/resident myelo-monocytes (Ly6C^lo/-^ GR1^lo/-^ CD11b^+^). Data are pooled from three independent experiments, with each individual dot representing one mouse. Horizontal lines represent the sample mean. See also S3 Fig. **(B)** Giemsa-stained infected bone marrow monocytes, myeloblast-like cells and macrophages bearing intracellular Leishmania amastigotes (100 X under oil immersion lens). Scale bar = 5μm. **(C)** Sca-1 and MHC-II expression on Ly6C^hi^ and Ly6C^lo/-^ monocyte subsets. **(D)** Galectin and Ly6C expression (MFI) on Ly6C^hi^ monocytes. All bar graphs represent mean + SEM with 4 mice per group coming from one single infection. Similar results were obtained in a second, independent experiment. *P<0.05; **P<0.01; ***P<0.001.

GMPs as a population give rise to both granulocytes (mostly neutrophils) and monocytes. During resting hematopoiesis, approximately half of the bone marrow myeloid cells are granulocytes (SSC^hi^GR1^hi^Ly6G^+^), with the remaining half divided into Ly6C^hi^ monocytes ready to enter the circulation and a Ly6C^lo/-^ population that comprises alternative monocytes and macrophages in addition to immature stages of both monocytes and granulocytes. Ly6C^hi^ monocytes represented the only myeloid cell type whose proportion steadily increased over time in the infected marrow (Fig 3A). On day28, we observed infected cells with monocyte as well as myeloblast morphologies in bone marrow smears (Fig 3B). All monocytes contained a single parasite, while the myeloblast-like cells, similar to mature macrophages, could be found to house several. These latter, productively infected cells could represent either immature myeloid cells or potentially an intermediate stage in monocyte maturation towards a more macrophage-like morphology.

To better evaluate monocyte differentiation and maturation, we sought to determine if the newly generated monocytes had acquired an altered, regulatory phenotype, similar to what has been reported in response to trypanosomal infection [25]. Ly6C^hi^ monocytes rapidly acquired Sca1 expression, similar to the emergency GMPs (Fig 3C and S3 Fig). In comparison, only a small percentage of Ly6C^lo/-^ cells upregulated Sca1. Ly6C^hi^ monocytes also upregulated MHCII (Fig 3C and S3 Fig) and down-regulated Ly6C expression (Fig 3D), which suggested that they had been exposed to IFN-γ [25]. To further support the hypothesis of monocyte developmental skewing, the Ly6C^hi^ monocytes generated in *L*. *donovani*–infected mice also upregulated Galectin-3 (Fig 3D and S3 Fig), which is associated with alternative macrophage activation [26], IL-10 production [27], and pro-fibrotic responses. These data collectively show that *L*. *donovani* subverts bone marrow hematopoiesis, enhancing the generation of emergency GMPs and the differentiation of monocytes that, due to their regulatory or immature phenotype, could represent safe targets and promote parasite expansion [22].

### Fzd6 promotes bone marrow response to *L*. *donovani*

**Fig 4:**
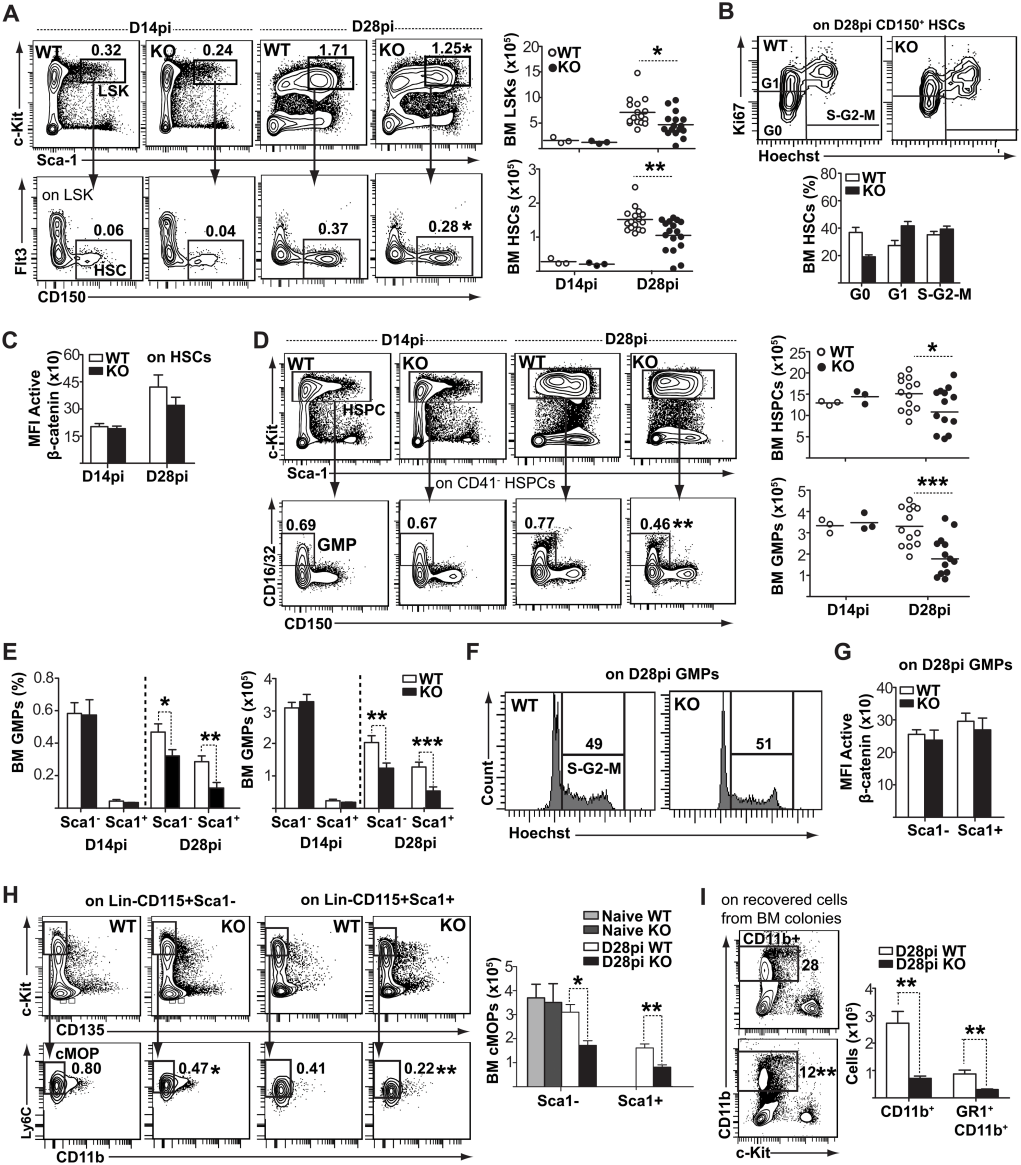
Frizzled-6 is required for parasite-induced expansion and myeloid differentiation of HSPCs. **(A)** Analysis of bone marrow LSK and HSC compartments in infected *Fzd6*^-/-^ (KO) and *Fzd6*^+/+^ (WT) mice. Mean percentage for LSKs and HSC-like cells within total bone marrow are indicated in the corner of each histogram. Graphs show the numbers of LSKs and HSCs on days 14 and 28. Day 28 data are pooled from three independent experiments, with each individual dot representing one mouse. Horizontal lines represent the sample mean. **(B)** Representative flow-cytometry plots for cell cycle analysis of WT and KO HSCs on day 28. Graph shows percent CD150^+^ HSC like cells in the G0, G1 and S-G2-M phases of cell cycle. **(C)** Intracellular active β-catenin levels (MFI) in WT and KO CD150^+^ HSCs on days 14 and 28. **(D)** Flow cytometry analysis of BM GMPs. Numbers in the histograms represent mean percentage for WT and KO GMPs. Graphs show the numbers of HSPCs and GMPs at day 14 and 28. Day 28 data are pooled from three independent experiments, with each individual dot representing one mouse. See also S4, S5 and S6 Figs. **(E)** Percentage and numbers of Sca-1^-^ and Sca-1^+^ GMPs on day 14 and 28 pi. **(F)** Cell cycle analysis of WT and KO GMPs on day 28. Similar results were obtained from six individual mice for each group. **(G)** Intracellular active β-catenin levels (MFI) in different GMP subsets on day 28. **(H)** Gating strategy and representative flow cytometry plots for common monocyte progenitors (cMoPs). BM cells were first gated on Lin^-^ (CD3ε^−^B220^−^NK1.1^−^Ly6G^−^) CD115^+^ and then subdivided according to Sca1. cMoPs were identified as cKit^+^CD135^-^Ly6C^+^CD11b^-^ within Lin^-^ CD115^+^ cell populations. Mean percentage for cMoPs within total bone marrow is depicted in flow cytometry plots, and the graph shows absolute numbers (mean + SEM from six mice per group). To note, there were no Sca1^+^ cMoPs in naïve mice. **(I)** Flow cytometry analysis of cells recovered from myeloid colony forming assays. Numbers shown in different quadrants indicate the mean percentage in CD11b^+^ cells. Histogram represents pooled data from one single infection for a total of five mice. Similar results were obtained in a second, independent experiment. All bar graphs represent mean + SEM with 10 mice per group for day 28 pooled from two independent experiments and 3 mice per group for day 14 unless otherwise noted. *P<0.05; **P<0.01; ***P<0.001.

We have previously shown that the absence of the Wnt signaling receptor Frizzled6 (Fzd6) dampens HSC expansion during LPS-induced emergency hematopoiesis [15]. Given that Wnt signaling was induced in *L*. *donovani*–activated HSCs (Fig 1H), we sought to evaluate if Fzd6 was also important for the bone marrow response to the parasite, and if we could use Fzd6-deficient mice as a tool to investigate the importance of the bone marrow response in promoting parasite expansion. There was no significant difference in bone marrow LSK or HSC numbers between *Fzd6*^*-/-*^ and *Fzd6*^*+/+*^ mice on day 14, before the expansion (Fig 4A) or on day 21 (S4 Fig). However, expansion was blunted in *Fzd6*^*-/-*^ mice on day 28 post-infection (Fig 4A), which corresponds to the peak of expansion (Fig 1G). The difference in HSPC accumulation was not due to decreased proliferation, as *Fzd6*^*-/-*^ HSCs entered the cell cycle at least as efficiently as their *Fzd6*^*+/+*^ counterparts (Fig 4B). If anything, there was a decrease in the proportion of quiescent HSCs in *Fzd6*^*-/-*^ bone marrow on day 28 (Fig 4B), correlating with their previously reported self-renewal defect [15], and indicating that the *Fzd6*^*-/-*^ bone marrow responded to the infection. However, the response did not result in as great an accumulation of HSPCs. There was also no significant difference in β-catenin activation (Fig 4C), similar to what we had previously reported at steady state [15]. These results suggested that *Fzd6*^*-/-*^ mice could provide a model to investigate the impact of *L*. *donovani*–adapted hematopoiesis on parasite expansion.

*Fzd6*^*-/-*^ mice display no specific defects in the bone marrow MPC compartment at steady state (S5 Fig) or on day 14 post-infection, prior to the parasite-induced changes in hematopoiesis (Fig 4D and 4E). However, on day 28 we observed a two-fold decrease in GMPs (Fig 4D) and more specifically in Sca1^+^ emergency GMPs (Fig 4E). Similar to HSCs, there was no difference in cell cycle (Fig 4F) or β-catenin activity (Fig 4G) between *Fzd6*^*-/-*^ and *Fzd6*^*+/+*^ GMPs; nor was GM-CSF receptor expression altered (S6 Fig). We also evaluated the presence of common monocyte precursors (cMoPs; Lin^-^CD115^+^cKit^hi^Flt3^-^Ly6C^+^CD11b^-^, S5 Fig), and found that the number of cMoPs was slightly higher in *L*. *donovani*–infected bone marrow when compared to uninfected controls (Fig 4H). cMoPs acquired Sca1 expression in the infected marrow, similar to GMPs and Ly6C^hi^ monocytes (Figs 2D, 2E and 3C). However, *Fzd6*^*-/-*^ cMoPs were not expanded in response to *L*. *donovani* and a lower proportion had acquired Sca1 (Fig 4H). Myeloid colony formation in culture was also decreased, with HSPCs isolated from infected *Fzd6*^*-/-*^ bone marrow generating 3-times fewer CD11b^+^ myeloid cells than *Fzd6*^*+/+*^ controls (Fig 4I). Together these data show that the presence of Fzd6 promotes parasite-induced HSPC expansion and skewing towards an emergency phenotype.

### Enhanced myelopoiesis correlates with increased parasite burden

**Fig 5:**
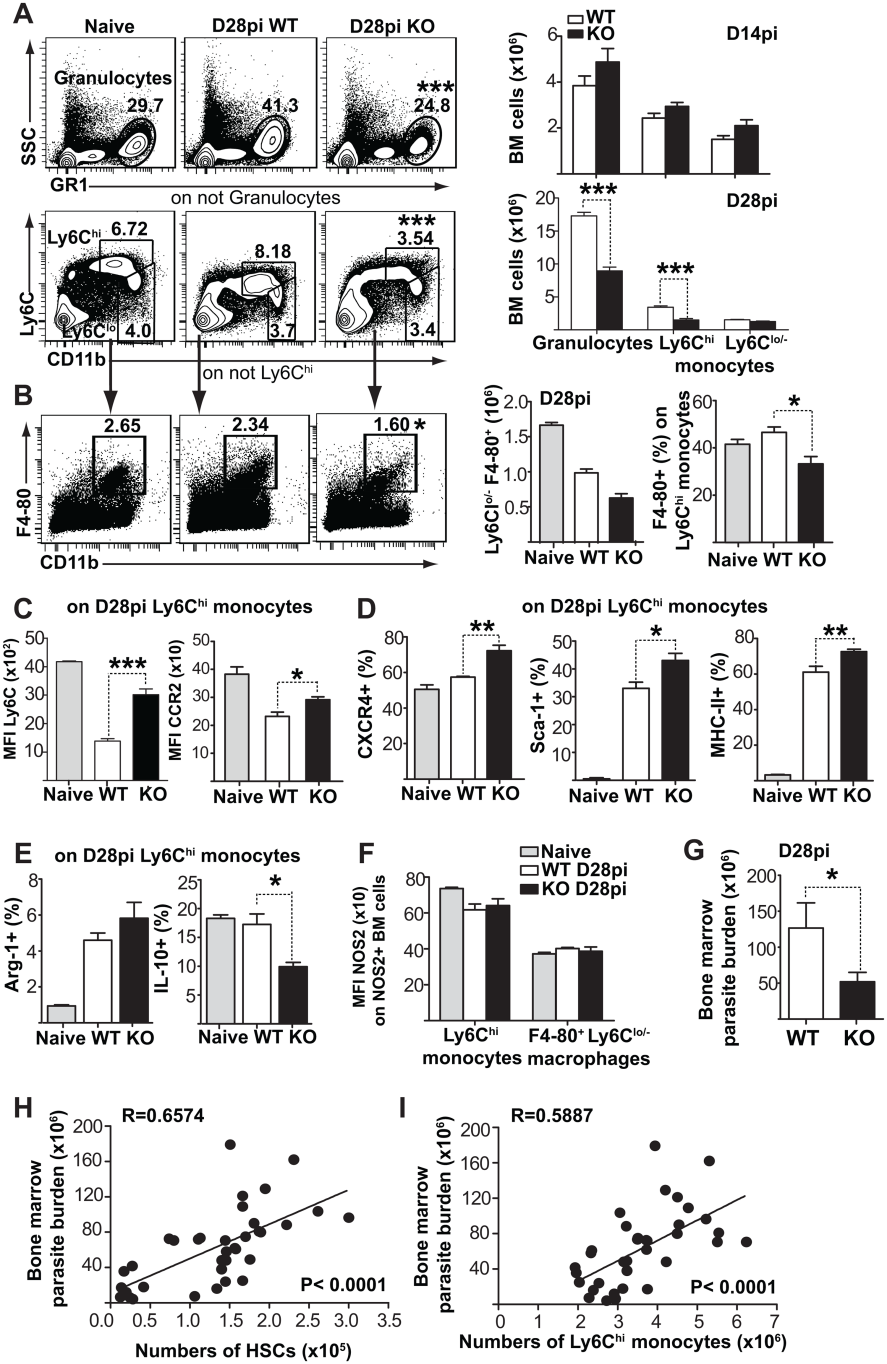
Diminished myeloid output in *Fzd6*^-/-^ mice correlates with a reduced parasite burden during the chronic phase of infection. **(A)** Analysis of bone marrow myeloid subsets infected *Fzd6*^-/-^ (KO) and *Fzd6*^+/+^ (WT) mice. Mean percentage for each cell subset is indicated within flow cytometry plots. Graphs show numbers of granulocytes and monocytes on day 14 and 28. See also S4, S6 and S7 Figs. **(B)** Numbers within flow cytometry plots indicate mean percentage of Ly6C^lo/-^ F4-80^+^ bone marrow macrophages. Histograms show total numbers of macrophages and percent F4-80^+^ within Ly6C^hi^ monocytes (mean + SEM from seven mice per group). **(C)** Ly6C and CCR expression (MFI) on Ly6C^hi^ monocytes at day 28pi. **(D)** Percentage of CXCR4^+^, Sca-1^+^ and MHC-II^+^ cells within Ly6C^hi^ monocytes on day 28pi. See also S8 Fig. **(E)** Percentage of Arginase-1 (Arg-1) and IL-10 expressing cells within Ly6C^hi^ monocytes. **(F)** NOS2 expression (MFI) on Ly6C^hi^ monocytes and Ly6C^lo/-^ F4-80^+^ bone marrow macrophages at day 28pi. **(G)** Parasite burden determined by the limiting dilution assay in WT and KO bone marrow at day 28. Data shown were pooled from two independent infections with 10 mice per genotype. **(H-I)** Pearson's correlation coefficient was used to assess correlation between bone marrow parasite burden and bone marrow HSCs **(H)** and Ly6C^hi^ monocytes **(I)** during the course of the infection. Data for correlation were pooled from C57BL/6, *Fzd6*^+/+^ and *Fzd6*^-/-^ mice at day 14, 21 or 28. All bar graphs represent mean + SEM with 6 mice per group for day 28 and 3 mice per group for day 14 unless otherwise noted. Similar results were obtained from three independent experiments for day 28. *P<0.05; **P<0.01; ***P<0.001.

To further investigate the impact of HSPC expansion on downstream myeloid differentiation during infection, we evaluated the acquisition of regulatory markers by *Fzd6*^-/-^ Ly6C^hi^ monocytes. There was no difference in the proportion of granulocytes (SSC^hi^ GR1^hi^/Ly6G^+^), mature Ly6C^hi^ monocytes, or Ly6C^lo^ monocytes/macrophages at steady state (S7 Fig) or on day 14 (Fig 5A) or day 21 (S4 Fig). However, there was a specific decrease in the output of both GR1^hi^ and Ly6C^hi^ myeloid cells (Fig 5A) and F4-80^+^ CD11b^int^ macrophages (Fig 5B) in the *Fzd6*^-/-^ bone marrow on day 28. In contrast to *Fzd6*^+/+^ cells, *Fzd6*^-/-^ Ly6C^hi^ monocytes did not downregulate Ly6C or Ccr2 (Fig 5C and S8 Fig) and expressed higher levels of Cxcr4 (Fig 5D and S8 Fig). Ccr2 and Ly6C downregulation are generally associated with monocyte differentiation into macrophages [28–30], while Cxcr4 expression has been recently shown to correspond to a transitional stage between cMoPs and mature monocytes [31]. Together, these data suggest that *Fzd6*^-/-^ Ly6C^hi^ monocytes were more immature and perhaps less likely to differentiate locally into macrophages.

Both *Fzd6*^-/-^ and *Fzd6*^+/+^ Ly6C^hi^ monocytes expressed elevated Sca1 and MHCII (Fig 5D and S8 Fig), indicative of an inflammatory microenvironment. In further support of their potential regulatory function, Ly6C^hi^ monocytes from both groups upregulated Arginase1 expression, and expressed low levels of IL-10 (Fig 5E and S8 Fig). Furthermore, there was no upregulation of iNOS by Ly6Chi monocytes or F4-80^+^ bone marrow macrophages from either *Fzd6*^-/-^ or *Fzd6*^+/+^ mice (Fig 5F and S8 Fig). Thus, *Fzd6*^-/-^ Ly6C^hi^ monocytes are produced in lower numbers, presumably due to decreased expansion of upstream progenitors, but they do not significantly differ in the expression of activation markers, such as MHCII, Sca1, or iNOS. However, *Fzd6*^-/-^ mice present with lower parasite burden in the bone marrow (Fig 5G), suggesting that the decreased production of “safe targets” in the form of Ly6C^hi^ monocytes might be sufficient to dampen parasite expansion, independent of their functional capacity. In further support of this hypothesis, we established a statistical correlation between bone marrow parasite burden and the number of CD150^+^ HSCs (Fig 5H) as well as bone marrow Ly6C^hi^ monocytes (Fig 5I), independent of mouse genotype or the stage of infection (D14-D28).

**Fig 6:**
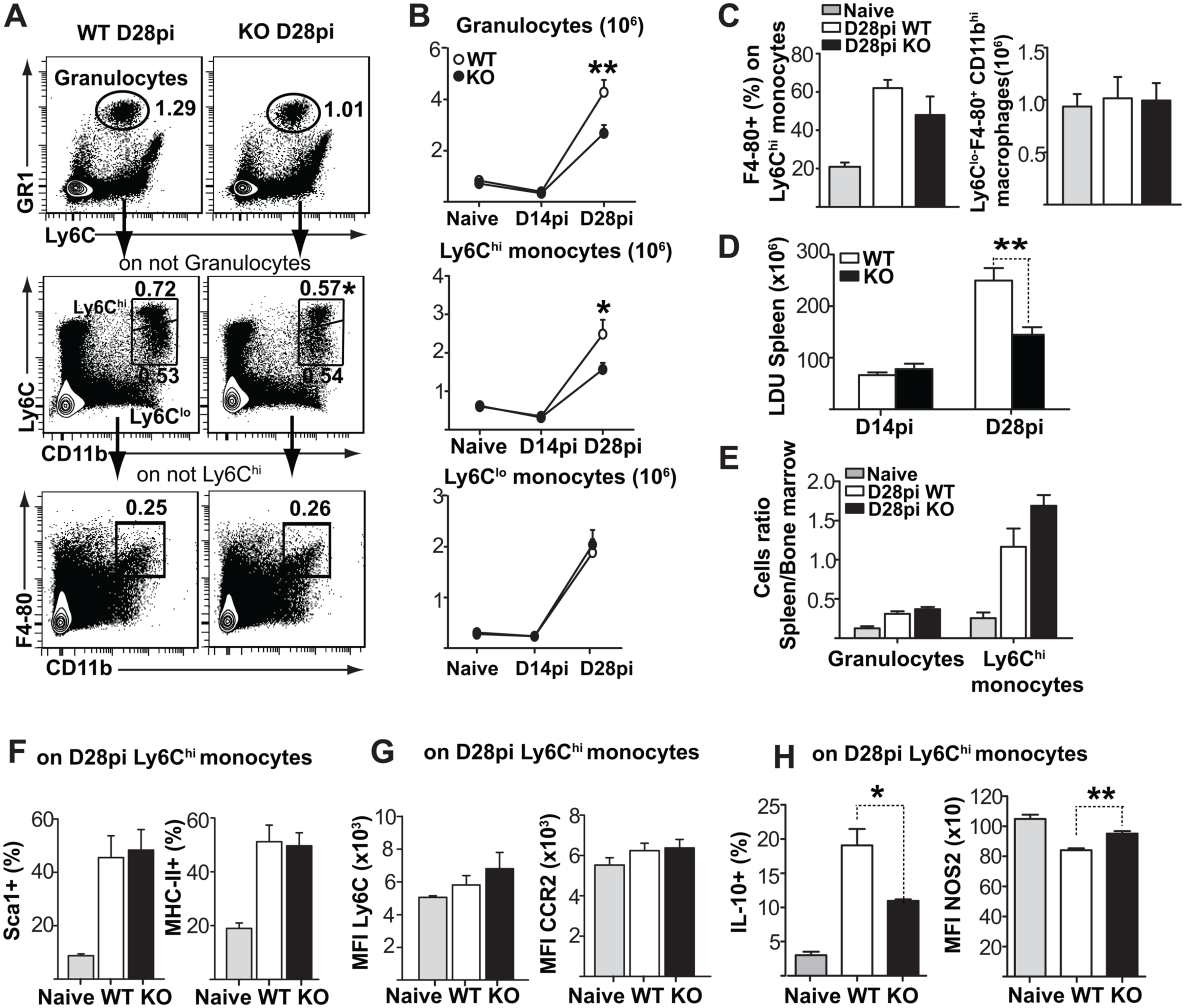
Decreased accumulation of myeloid cells is accompanied with reduced parasite burden in *Fzd6*^-/-^ spleen. **(A)** Analysis of myeloid subsets in the spleen of infected *Fzd6*^-/-^ (KO) and *Fzd6*^+/+^ (WT) mice. Mean percentage for each cell subset is indicated within flow cytometry plots. See also S7, S8 and S9 Figs. **(B)** Graphs show numbers of granulocytes and monocytes on day 14 and 28. **(C)** F4-80^+^ cells within Ly6C^hi^ monocytes and numbers of Ly6C^lo/-^ F4-80^+^ macrophages in the spleen. **(D)** Parasite burden expressed as Leishmania Donovani Units (LDU) in spleen on days 14 and 28pi. **(E)** Ratio of splenic to bone marrow granulocytes and Ly6C^hi^ monocytes in infected KO and WT mice on day 28 (mean + SEM from six mice per group). **(F)** Percentage of Sca-1^+^ and MHC-II^+^ cells within Ly6C^hi^ monocytes on day 28pi. **(G)** Ly6C and CCR2 expression (MFI) on Ly6C^hi^ monocytes at day 28pi. **(H)** Percentage of IL-10 expressing cells and NOS2 expression (MFI) on Ly6Chi monocytes at day 28pi. All bar graphs represent mean + SEM with 18 mice per group for day 28 pooled from three independent experiments and 3 mice per group for day 14 unless otherwise noted. *P<0.05; **P<0.01; ***P<0.001.

To determine whether the differences in bone marrow were also reflected in peripheral organs, we evaluated the recruitment of myeloid cells also to liver and spleen, two major target organs in visceral leishmaniasis. Similar to the bone marrow, spleen myeloid compartment was still unchanged on day 14, but the numbers of granulocytes and monocytes were all substantially increased by day 28 (Fig 6A and 6B). Once more, there was no difference between *Fzd6*^-/-^ and *Fzd6*^+/+^ mice at steady state (S7 Fig), or earlier during the infection, before the onset of altered hematopoiesis (Fig 6B and S4 Fig). We observed a specific decrease in *Fzd6*^-/-^ GR1^hi^ and Ly6C^hi^ myeloid cells (Fig 6A and 6B) with no difference in Ly6C^lo^ monocytes or F4-80^+^ CD11b^int^ red pulp macrophages (Fig 6A–6C). There was also a corresponding decrease in Ly6C^hi^ monocytes in the liver (S9 Fig). The decrease in myeloid cells correlated with decreased parasite burden on day 28 in *Fzd6*^-/-^ spleen (Fig 6D) and liver (S9 Fig). Wnt signaling has been reported to affect leukocyte infiltration [18]; however, there was no difference in the proportion of Ly6C^hi^ monocytes in peripheral blood (S4 Fig) or in the relative ratio of Ly6C^hi^ monocytes present in spleen as compared to bone marrow (Fig 6E), suggesting that the decrease in *Fzd6*^-/-^ peripheral monocytes was mainly due to differences in hematopoiesis. There was no difference in the expression of Sca1, MHCII, Ly6C, or Ccr2 between *Fzd6*^-/-^ and *Fzd6*^+/+^ Ly6C^hi^ monocytes in spleen (Fig 6F and, 6G) or liver (S9 Fig). *Fzd6*^+/+^ Ly6C^hi^ monocytes expressed IL-10 (Fig 6H and S8 Fig) but did not upregulate iNOS (Fig 6H), thus supporting the theory that they may have regulatory functions. A smaller proportion of *Fzd6*^-/-^ Ly6C^hi^ monocytes were IL-10^+^. Wnt5a has been reported to directly induce IL-10 production in dendritic cells through a non-canonical pathway [32], while β-catenin activation promotes TLR-mediated IL-10 secretion by human monocytes [33]. Overall these results suggest that the decreased monocyte output by the *Fzd6*^-/-^ bone marrow translates into decreased monocyte numbers in the periphery, decreased IL-10 production, and an apparently improved control of parasite expansion.

### Decreased parasite expansion in *Fzd6*^-/-^ mice is not due to enhanced T lymphocyte activity

**Fig 7:**
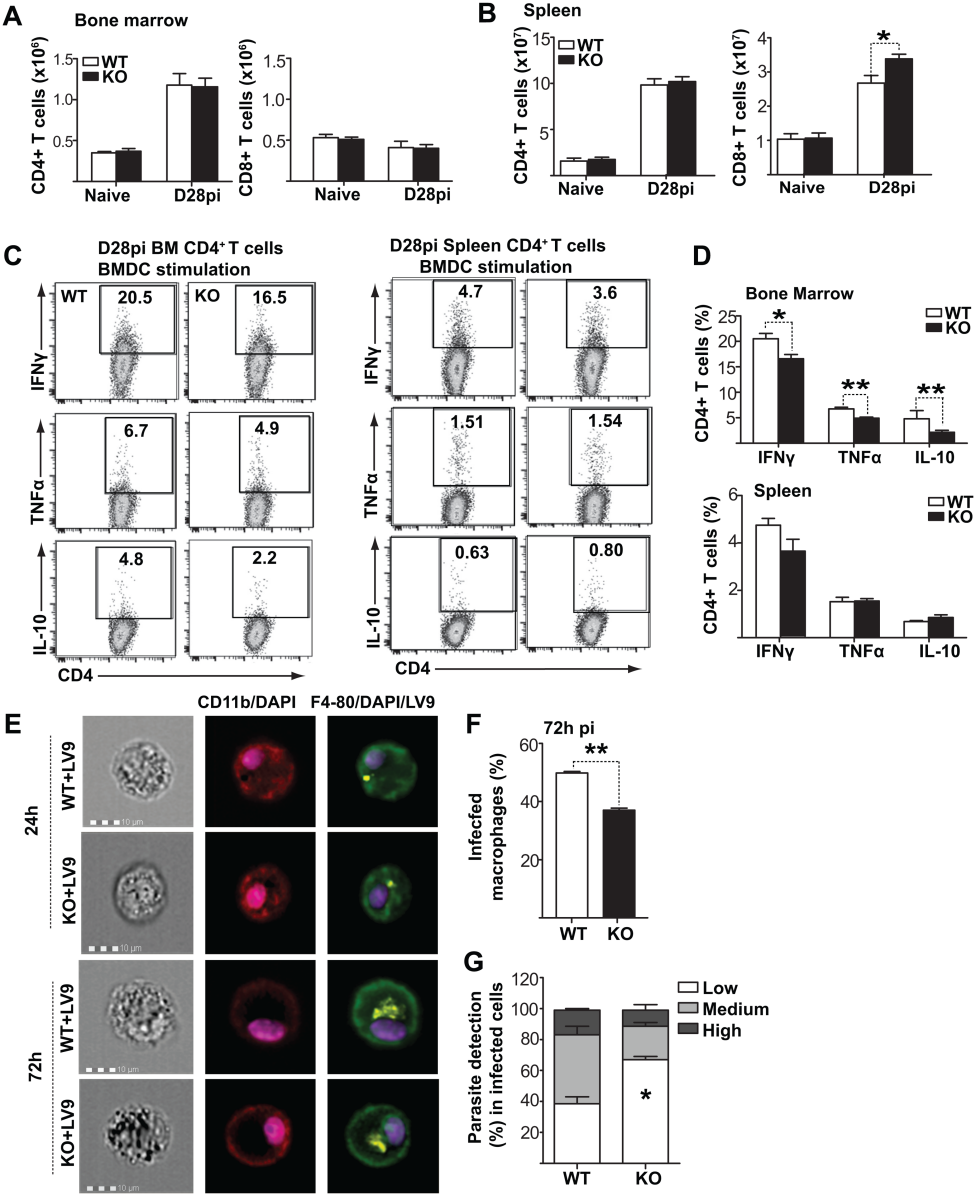
*Fzd6*^-/-^ T lymphocytes are functionally indistinguishable from their *Fzd6*^+/+^ counterparts. **(A-B)** Numbers of CD4^+^ and CD8^+^ T cells in BM **(A)** and spleen **(B)** of naïve and infected mice on day 28. **(C-D)** Cytokine production by bone marrow and spleen CD4^+^ T cells isolated from infected mice and stimulated *ex vivo* with parasite-pulsed bone marrow dendritic cells. Representative flow cytometry data are shown in **(C)**. Graph in **(D)** shows compiled results from a representative experiment. See also S10 Fig. All bar graphs represent mean + SEM with 7 mice per group for day 28. **(E)** Macrophages derived from naïve *Fzd6*^-/-^ (KO) and *Fzd6*^+/+^ (WT) bone marrow were either left untreated, stimulated with IFN-γ alone or first primed with IFN-γ and then infected with PKH26-labeled *L*. *donovani* amastigotes. See also S10 Fig. Imaging flow cytometry analysis of macrophages infected with fluorescent *Leishmania* parasites, showing multiple parasites within both *Fzd6*^-/-^ (KO) and *Fzd6*^+/+^ (WT) macrophages. **(F)** Histograms represent percentage of infected macrophages and **(G)** parasite numbers in infected macrophages 72h post-infection. Low = 1–3, medium = 4–10, high = >10 parasites / cell. Bar graphs represent mean + SEM from two independent experiments.

Improved T lymphocyte function [34–37] or macrophage-mediated parasite killing [38] could also contribute to diminished parasite expansion, independent of changes in myeloid differentiation. We first investigated T lymphocyte recruitment to the bone marrow and their ability to produce cytokines. There is no major difference in T lymphocyte development in the absence of Fzd6 [15]. There was also no major difference in the numbers of T lymphocytes in the bone marrow or spleen of *Fzd6*^-/-^ mice when compared to controls (Fig 7A and 7B and S10 Fig), as we observed a similar increase in CD4^+^ T lymphocytes in both groups. There was a slight but statistically significant increase in CD8^+^ T lymphocytes in the *Fzd6*^-/-^ spleen. However, CD8^+^ T cells become progressively exhausted in chronic visceral leishmaniasis [39]. There was also no increase in cytokine production by either bone marrow or splenic T lymphocytes in response to parasite presented by bone marrow-derived dendritic cells (Fig 7C and 7D), indicating that T lymphocytes were unlikely to explain the difference in parasite burden.

To investigate the contribution of macrophages, we first generated bone marrow-derived macrophages (BMDMs) in culture from *Fzd6*^-/-^ and *Fzd6*^+/+^ progenitors. We had not observed any striking differences in the Ly6C^lo^ CD11b^+^ population that includes bone marrow macrophages at steady state (S7 Fig), although the F4-80^+^ CD11b^int^ macrophage population is decreased in mice infected with *L*. *donovani* (Fig 5A and 5B). Similarly, there was no difference in the expression of CD11b, F4/80, MHCII or Sca1 between *Fzd6*^-/-^ and *Fzd6*^+/+^ macrophages at baseline (S10 Fig). There was also no difference in their upregulation of MHCII in response to IFNγ (S10 Fig), and we detected no significant changes in parasite uptake using fluorescence-labeled amastigotes (Fig 7E and S10 Fig). However, there was an improvement in parasite control 72h post-infection in the presence of IFNγ (Fig 7F and 7G), suggesting that *Fzd6*^-/-^ bone marrow macrophages could partially contribute to the decreased parasite burden. Interestingly, exposure to the parasite resulted in massive upregulation of Sca1 on BMDMs (S10 Fig), while IFN-γ alone had no impact. Sca1 expression in the Ly6C^lo^ bone marrow population could therefore represent a biomarker for active infection rather than the IFN-dependent acquisition of a regulatory phenotype.

### Bone marrow cytokine environment promotes the generation of permissive monocytes

**Fig 8:**
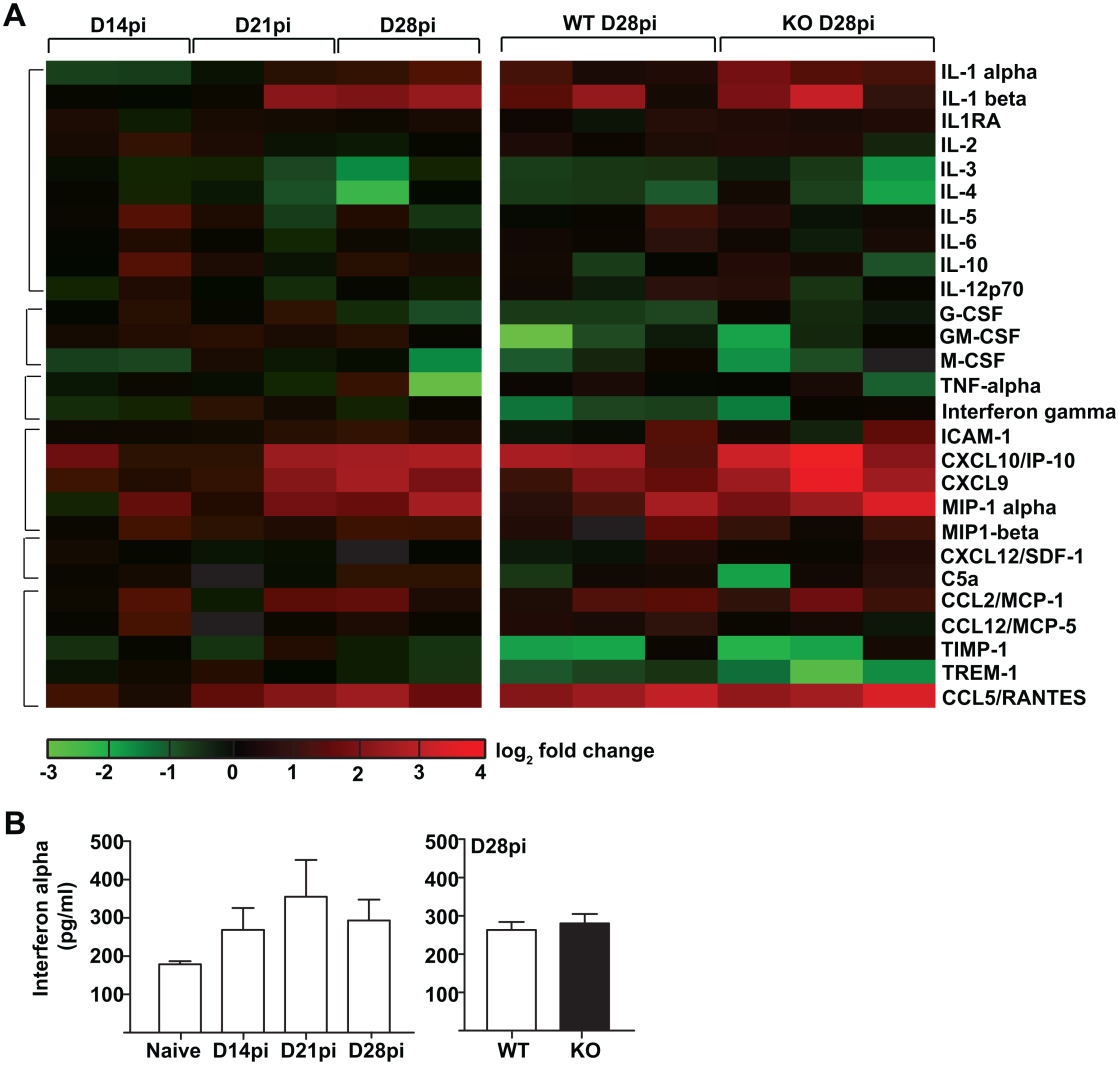
*Fzd6*^-/-^ bone marrow microenvironment is enriched in pro-inflammatory cytokines and chemokines. **(A)** Fold change in cytokine/chemokine levels in *Fzd6*^-/-^ (KO) and *Fzd6*^+/+^ (WT) bone marrow extracellular milieu on day 28pi as compared to untreated mice. Heatmap represents log 2 fold change in mean pixel density per cytokine/chemokine in at various time points of infection as compared to untreated mice. Each square represents one individual experiment coming from a pooled sample of 4–6 mice per group. **(B)** Interferon alpha levels in bone marrow supernatant obtained from naïve and infected mice.

*L*. *donovani*-infected macrophages produce TNF-α and GM-CSF, which have been suggested to promote myelopoiesis [12]. However, stromal macrophages represent a relatively small proportion of all bone marrow cells, with the vast majority of the marrow being occupied by the developing myeloid, lymphoid and erythroid cells. To obtain a more comprehensive picture of the bone marrow cytokine environment during infection, we used the supernatant from freshly harvested total bone marrow cells as a surrogate for the extracellular milieu and compared uninfected mice to *L*. *donovani*-infected mice at different time points. The levels of MIP1α/Ccl3, IL-1, and IFNγ-inducible factors, such as ICAM-1, IP10/Cxcl10 and Cxcl9 were all increased in the chronic phase of the infection (Fig 8A). Both IFN-α and IFN-γ were also upregulated on day 21 (Fig 8A and 8B), at the beginning of the chronic phase, and could thus contribute to HSPC expansion in the bone marrow [4, 7, 24, 40]. However, IFN-γ levels appeared to decrease back to or even below baseline levels by day 28. There was no significant difference in the levels of IFN-γ, IFN-α, or myeloid growth factors GM-CSF, M-CSF, IL-3, or IL-6 between *Fzd6*^-/-^ and *Fzd6*^+/+^ mice. However, *Fzd6*^-/-^ bone marrow environment demonstrated an even more dramatic upregulation of MIP1α and IL-1, together with a stronger IFNγ response. These observations together with data from Figs 4 and 5 suggest that the reduced parasite burden in *Fzd6*^-/-^ mice is at least partially the result of a different inflammatory microenvironment that could contribute to its own maintenance via altered myeloid differentiation.

**Fig 9:**
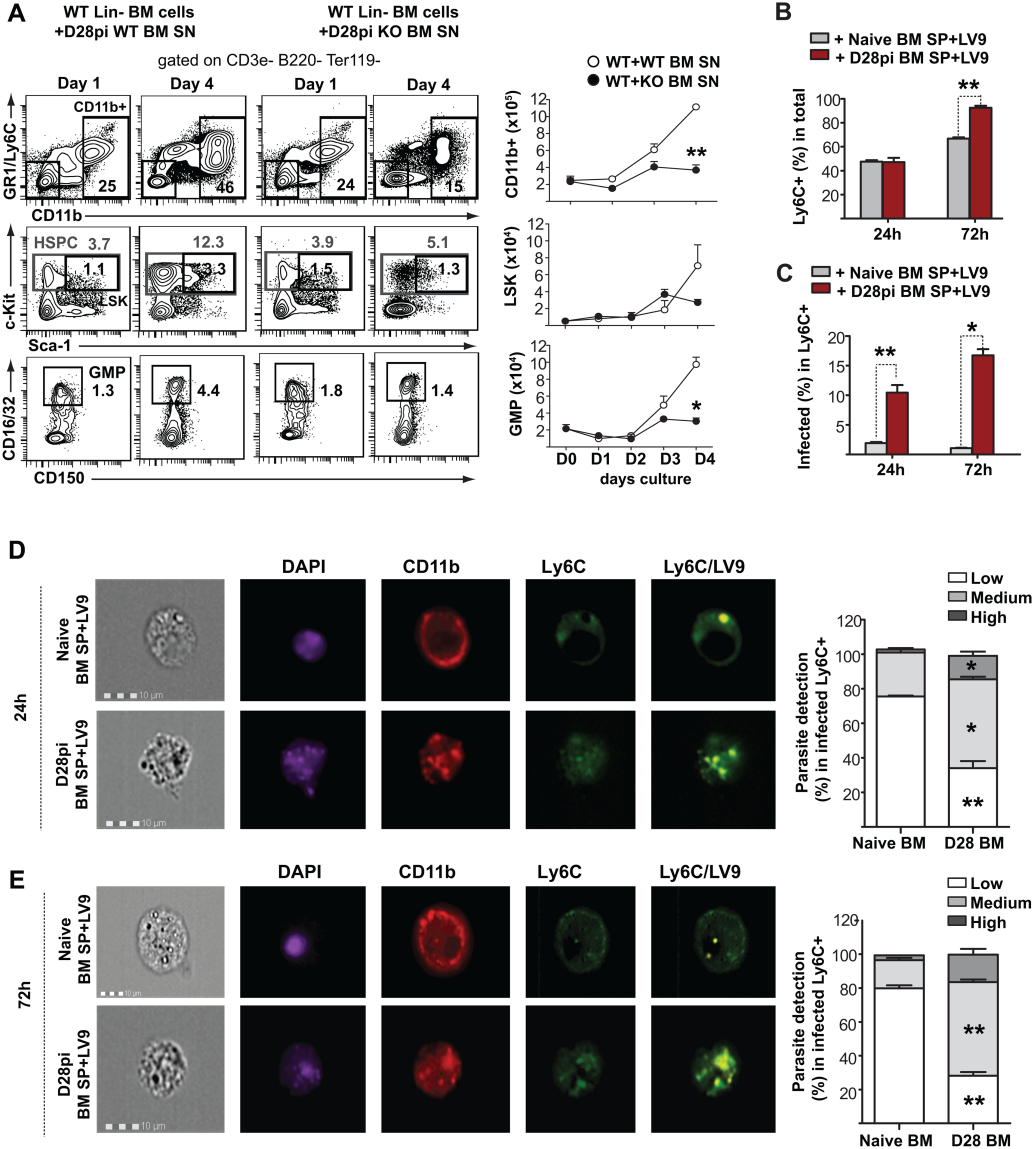
Infected bone marrow microenvironment directly promotes HSPC expansion and the generation of permissive monocytes. Freshly isolated lineage-depleted *Fzd6*^+/+^ (WT) BM cells were cultured in complete medium supplemented with 30% BM supernatant as indicated. **(A)** Representative flow cytometry data show the gating strategy for CD11b^+^, LSK and GMP populations. Graphs show numbers of cell recovered per 5x10^5^ cells seeded for each subset. Lin^-^ *Fzd6*^+/+^ (WT) BM cells cultured with BM supernatant obtained from infected *Fzd6*^-/-^ (KO) and *Fzd6*^+/+^ (WT) mice. See also S11 Fig. Data were pooled from three independent experiments. **(B)** Percentage of Ly6C^+^ monocytes in differentiation cultures following infection on day 4, and **(C)** the proportion of infected Ly6C^+^ monocytes after 24h and 72h. **(D-E)** Imaging flow cytometry analysis of monocytes infected with fluorescent Leishmania parasites, showing multiple parasites at **(D)** 24h and **(E)** 72h post-infection. Histograms represents parasite uptake in infected macrophages. Low = 1–3, medium = 4–10, high = >10 parasites respectively. Bar graphs represent mean + SEM from three independent experiments. *P<0.05; **P<0.01.

To investigate this hypothesis in culture, we used the bone marrow supernatants as source of growth factors and evaluated the expansion and differentiation of both *Fzd6*^-/-^ and *Fzd6*^+/+^ bone marrow progenitor cells over four days. Supernatants from both *Fzd6*^-/-^ and *Fzd6*^+/+^ infected bone marrow stimulated the emergence of GR1^+^/Ly6C^hi^/CD11b^+^ cells (Fig 9A and S11 Fig); however, their numbers were significantly lower in cultures that had been exposed to the *Fzd6*^-/-^ environment. Similar results were obtained also with GMPs and LSKs, indicating that the cytokine environment present in the *Fzd6*^-/-^ bone marrow significantly reduces HSPC expansion. The differentiation patterns were very similar when *Fzd6*^-/-^ progenitor cells were used (S11 Fig), suggesting that the initial steps of the progenitor cell response were not affected, similar to what we observed *in vivo* on day 14 (Fig 4A–4E), or over the first few days after transplant [15]. Cell-intrinsic self-renewal defects would only appear after a much longer period. Thus, although *L*. *donovani* induces the production of factors that promote myeloid differentiation in both *Fzd6*^-/-^ and *Fzd6*^+/+^ bone marrow, the *Fzd6*^-/-^ cytokine environment reduces HSPC expansion, possibly via myelosuppressive factors, which in turn results in the generation of fewer GR1^+^/Ly6C^hi^ monocytes both in culture (Fig 9) and *in vivo* (Fig 5).

To directly test whether the monocytes generated in the infected bone marrow environment were functionally different from normal in vitro—differentiated monocytes, we exposed the cultures to fluorescence-labeled *L*. *donovani* amastigotes. Less than 5% Ly6C^hi^ monocytes generated in the naïve setting became infected and there was no increase in the proportion of infected cells from 24h to 72h (Fig 9C–9E), although the proportion of monocytes containing efficiently replicating parasites increased over that period. In contrast, there was a 10-fold increase in the proportion of infected monocytes derived in the presence of supernatants coming from the infected bone marrow (Fig 9C–9E), and this proportion increased over time. In both cultures, the proportion of Ly6C^hi^ cells increased between 24h and 72h (Fig 9B), which could indicate either continued differentiation or the loss of Ly6C^lo/-^ cells due to cell death or increased adhesion. In summary, these results show that the *L*. *donovani*–infected bone marrow environment not only promotes myelopoiesis but also renders the newly generated cells more permissive to infection.

## Discussion

We have examined here the role of HSPC expansion and altered myeloid differentiation in promoting parasite proliferation during experimental visceral leishmaniasis. We demonstrate that *L*. *donovani* induces HSPC proliferation in the bone marrow, and promotes the generation of Sca1^+^ emergency GMPs and their differentiation into Ly6C^hi^ monocytes expressing regulatory markers, such as Sca1, MHCII, Galectin-3 and IL-10. In contrast, an inefficient emergency response and diminished accumulation of emergency GMPs and Ly6C^hi^ monocytes in *Fzd6*^-/-^ mice corresponds to lower parasite expansion in bone marrow and in the periphery. We further show that the bone marrow cytokine environment is sufficient to promote HSPC expansion and myeloid differentiation, and to render the newly produced monocytes more susceptible to infection. Collectively our data support the hypothesis that *L*. *donovani* modulates the host hematopoietic program to support its own expansion.

*Leishmania* parasites have developed multiple mechanisms to survive inside their classical targets, macrophages [38], and to promote the recruitment of more monocytes to the site of infection [23]. *Leishmania* infection has also been associated with increased extramedullary myelopoiesis in the spleen [8, 11, 22], which has been proposed to generate “safe targets”, or permissive monocytes for the parasite to replicate in [22]. Thus the parasite could not only modify its target cells to its liking but also promote their generation. *L*. *donovani* establishes a chronic infection in the bone marrow [12], the predominant site of adult hematopoiesis, and chronic leishmaniasis is associated with pancytopenia and may ultimately lead to bone marrow failure [8, 9]. Despite all this, the role of bone marrow in the pathogenesis of visceral leishmaniasis remains obscure. We have now characterized in detail the changes to the bone marrow HSPC compartment during experimental visceral leishmaniasis, and demonstrate that HSC-like cells and emergency GMPs are substantially expanded during the early phases of chronic infection. Emergency GMPs are associated with superior proliferative potential when compared to their classical Sca1^-^ counterparts, and would thus result in the generation of more progeny in the periphery [24]. In addition, our data provide functional evidence that *L*. *donovani*–induced adaptations to hematopoiesis are indeed important for parasite expansion: bone marrow cytokine environment from infected mice was not only sufficient to induce HSPC expansion and myeloid differentiation in culture but also promoted the infection of newly generated monocytes. Conversely, parasite burden directly correlated with bone marrow HSPC and Ly6C^hi^ monocytes numbers. Thus put together, our data strongly support the hypothesis that infection-adapted emergency myelopoiesis directly promotes parasite expansion and the persistence of a chronic infection.

Monocytes have been proposed as safe targets for *L*. *major* [22], and alternative monocytes contribute to the pathogenesis of *L*. *brasiliensis* [23]; however, the role of monocytes in the pathogenesis of visceral leishmaniasis is not well established [10]. Development of liver granulomas and the clearance of *L*. *donovani* in the liver are dependent on incoming monocytes and can be promoted with GM-CSF [41, 42], but the contribution of monocytes to the progression of infection in bone marrow and spleen is not known. Our results suggest that in contrast to liver, the accumulation of Ly6C^hi/int^ monocytes in spleen and bone marrow is detrimental to the host, resulting in higher parasite burden. We observed an increased frequency of IL-10^+^ and Arginase1^+^ Ly6C^hi^ monocytes in *L*. *donovani*-infected mice with no upregulation of iNOS, suggesting that the myeloid cells adopt indeed an M2-like phenotype. This could be due to the different environmental factors that promote their conversion to a regulatory phenotype [25], permissive to infection but unable to kill the parasite.

Bone marrow cytokine environment was responsible for mediating HSPC expansion and myeloid differentiation in culture. Our finding correlates with previous reports, suggesting that infected macrophages produce myeloid growth factors, such as GM-CSF, in culture [12]. However, our approach takes into account not only the infected macrophages but also other components of the bone marrow microenvironment, including the immature myeloid cells that compose the bulk of the marrow. When comparing the cytokine and chemokine environment from infected mice to uninfected controls, we saw that GM-CSF levels were actually downregulated in chronic infection, which could correlate with decreased leishmanicidal activity [42]. We also saw a corresponding increase in GM-CSFR expression on the surface of both GMPs and more mature myeloid cells, suggesting decreased ligand availability. These data suggest that the impact of a chronic *in vivo* infection with *L*. *donovani* on cytokine production may be very different from what is seen in culture. They also emphasize the need for more in depth analysis of various microenvironmental niches, as the cytokines present in different organs or even between separate sites within the same organ may have opposing effects.

Chronic leishmaniasis is associated with anemia and pancytopenia, signs of diminished HSC function and bone marrow failure. We observed a decrease in SDF-1/Cxcl12 and an increase in MIP-1α/Ccl3 in the infected bone marrow environment. Cxcl12 is required for HSC maintenance in the bone marrow [43], and a decrease in Cxcl12 levels is associated with HSC activation and release to peripheral sites, for example, during acute bacterial infection [44, 45]. Conversely, Ccl3 has been shown to modify bone marrow HSC niches, thus promoting the maintenance of leukemic stem cells, for example, at the expense of normal HSCs [46, 47]. Ccl3 can also promote the settlement of macrophages to the bone marrow [48]. Together, the decrease in Cxcl12 and the concomitant increase in Ccl3 observed in the bone marrow of *L*. *donovani*–infected mice are coherent with HSC activation and a gradual loss of function, and represent potential targets for intervention to prevent bone marrow failure.

A better understanding of the signaling pathways underlying bone marrow alterations during visceral leishmaniasis may also result in the development of immunotherapies. For example, it should be possible to develop complementary therapies to be used in combination with parasitostatic treatment that would selectively promote the generation of monocytes capable of controlling and ultimately eliminating the parasite. Alternatively, it might be possible to interfere with monopoiesis in a short-term, preventive treatment, targeted for travellers visiting an endemic area. Lastly, long-term trained immunity [49–51] almost certainly involves epigenetic alterations at the HSPC level. Identifying the factors that stimulate HSPC expansion and the skewing of myeloid differentiation towards a permissive phenotype could thus pave the way for the design of vaccines dependent on innate immune cell function.

Taken together, we report here a substantial activation and accumulation of HSC-like cells and Sca1^+^ emergency GMPs during the early stages of chronic *L*. *donovani* infection, concomitant with the sudden increase in bone marrow parasite burden. We further demonstrate that reduced HSPC expansion is associated with lower parasitemia and thus provide mechanistic evidence that *L*. *donovani* indeed subverts bone marrow hematopoiesis *in vivo* to support its own expansion. Our results support the hypothesis that emergency hematopoiesis directly contributes to the pathogenesis of visceral leishmaniasis, and suggest that HSPCs could represent an interesting therapeutic target.

## Materials and methods

### Experimental animals and parasites

C57BL/6 mice were purchased from The Jackson laboratory (Bar Harbor, ME). Mice deficient in Frizzled 6 (*Fzd6*^−/−^) were first backcrossed to C57BL/6 for ten generations and then maintained under specific pathogen-free conditions in sterile ventilated racks at the animal facility of INRS-Institut Armand-Frappier (CNBE), as described [15]. Female *Fzd6*^-/-^ mice were compared to sex-matched *Fzd6*^+/+^ littermates unless otherwise noted. *Leishmania donovani* (strain LV9) were maintained by serial passage in *B6*.*129S7-Rag1tm1Mom* mice, and amastigotes were isolated from the spleens of infected animals. Experimental mice were infected by injecting 2×10^7^ amastigotes via the lateral tail vein. Bone marrow and splenic parasite burdens were determined either by limiting dilutions or by examining methanol-fixed, Giemsa stained tissue impression smears [52]. Data are presented as number of parasites per bone marrow (one femur and one tibia) or as Leishmani Donovan Units (LDU).

### Ethics statement

All procedures were in accordance with the Canadian Council on Animal Care guidelines and approved by the Comité institutionnel de protection des animaux of the INRS (CIPA #1411–02, 1210–06, 1510–02).

### Flow cytometry

Bone marrow was harvested by flushing tibiae and femora in phosphate-buffered saline (PBS), and the cells were then passed through a 25-gauge needle to obtain a single cell suspension. PBS was supplemented with 0.1% bovine serum albumin (BSA) and 0.5mM ethylene-diamine-tetra-acetic acid (EDTA) for flow cytometry staining. See S1 Table for a complete list of antibodies. For intracellular staining, surface stained cells were fixed and permeabilized using the Foxp3 staining kit (eBioscience, San Diego, CA) and then incubated with appropriate antibodies. For cell cycle analysis, cells were first incubated for 30 min at 37°C with Hoechst #33342 (Sigma-Aldrich, Oakville, ON, Canada) in DMEM supplemented with 10% Premium FBS (Wisent Bioproducts, St-Bruno, QC, Canada) and 1 mM HEPES (Life Technologies, Burlington, ON, Canada), followed by staining with surface antibodies and intracellular anti-Ki67 as described above. Samples were acquired with a four-laser LSR Fortessa flow cytometer (BD Biosciences, Mountain View, CA) and analyzed using BD FACS Diva software (BD Biosciences) or FlowJo (for histogram overlays; Tree Star).

### Wright-Giemsa staining

Freshly isolated bone marrow cells from infected mice were collected on slides using a cytospin centrifuge (Hettich, Tuttlingen, Germany) at 800rpm for 5 min. The smears were air dried and stained with modified Wright-Giemsa stain (Protocol Hema3; Thermo Fisher Scientific) as per manufacturer’s instructions. Slides were mounted using Fluoromount-G (SouthernBiotech) and coverslips sealed with nail polish. Analyses of infected cells were performed using a Nikon Eclipse E800 microscope (Nikon) with a 100x oil immersion lens and images were captured with a digital camera (COOLPIX 990; Nikon).

### Colony assays

Freshly harvested cells were seeded in duplicate into 35mm non-adherent petri dishes at a density of 10^4^ cells/dish in methylcellulose medium containing stem cell factor, IL-3, IL-6, and Erythropoietin (Methocult GF M3434, Stem Cell Technologies, Vancouver, BC, Canada). The cultures were incubated at 37°C in 5% CO2 for 10 days and hematopoietic colonies were identified based on morphology under an inverted microscope.

### T cell stimulation

To analyze endogenous CD4 T cell responses, bone marrow-derived dendritic cells (BMDCs) were pulsed with fixed parasites for 24 h at 37°C. Splenocytes or bone marrow cells from infected animals were then added to BMDCs and incubated for 2 h at 37°C. Brefeldin A (BD Pharmingen) was added for an additional 4 h, after which cells were stained with appropriate antibodies as described above.

### Macrophage infection and parasite survival

Macrophages were derived from total bone marrow cells in IMDM supplemented with 10% very low endotoxin FBS and 15% L929-cell conditioned medium [53]. Purity of macrophage cultures was determined by flow cytometry 7 days after culture. Macrophages were then plated 2-3x10^6^ cells per 35mm non-adherent petri dish and either left untreated or stimulated with 10ng/ml IFN-γ for 2h. IFN-γ-primed macrophages were infected with PKH26-labeled *L*. *donovani* amastigotes at MOI of 5:1 for 24h or 72h in the continued presence of IFN-γ. Macrophages were harvested from cultures and analyzed by flow cytometry. For imaging flow cytometry, cells were first surface-stained with anti-CD11b and anti-F4/80 and then fixed and counterstained with DAPI (Life Technologies). Samples were acquired with Amnis Imagestream Mark II imaging flow cytometer (EMD Millipore) and analyzed with IDEAS v6.1 software using spot count function.

### Bone marrow cytokine/chemokine analysis

Bone marrow cell supernatants were collected from naïve or *L*. *donovani* infected mice by harvesting cells from both hind legs into 2 ml PBS, followed by centrifugation. Supernatants were pooled from at least four mice per sample, and analyzed using a membrane-based Proteome profiler mouse cytokine/chemokine array kit (R&D Systems). Array images were further analyzed using the NIH ImageJ image analysis software. Samples were normalized by subtracting pixel intensities from negative controls, and the fold changes for infected mice were determined as a ratio over naive mice of the same genotype. Levels of mIFN-α were determined by using mouse IFN-alpha Platinum ELISA (eBioscience).

### *In vitro* differentiation assay

Bone marrow cells were first enriched for progenitor cells using a Mouse hematopoietic progenitor cell enrichment kit (StemCell Technologies, Vancouver, BC), and their purity was determined by flow cytometry. 5x10^5^ Lin- BM cells per condition were divided in ten wells of a non-adherent 96-well plate (BD Falcon), and cultured in IMDM supplemented with 10% very low endotoxin FBS (Wisent) and 30% bone marrow supernatant in the presence of 10ng/ml IL-3, IL-6 and SCF for up to 4 days. Cells were harvested as indicated and their number and differentiation stage were analyzed by flow cytometry. Culture-derived monocytes were infected with PKH26-labeled L. donovani amastigotes at MOI of 5:1 for 24h or 72h. Cells were surface-stained with anti-CD11b and anti-Ly6C and then fixed and counterstained with DAPI (Life Technologies) for analysis by imaging flow cytometry as detailed above.

### Statistical analysis

Statistical significance was determined using ANOVA (for multiple comparisons) or two-tailed student's t test (for comparing *Fzd6*^+/+^ and *Fzd6*^-/-^). P < 0.05 was considered significant.

## Supporting information

S1 Fig Analysis of hematopoietic stem/progenitor cells in the spleen (Related to Fig 1). **(A-B)** Representative flow cytometry plots and gating strategy for LSKs and HSCs in the bone marrow and spleen. Numbers represent the percentage in each population for one individual mouse on day 28. **(C)** Representative flow cytometry plots and gating strategy for LSKs and HSCs in spleens of infected mice at various time points during infection. Mean percentage for each cell subset is indicated in histograms. **(D)** Intracellular β-catenin expression on bone marrow HSC cell like subsets in naïve and infected mice at D28pi.
(TIF)

S2 Fig Analysis of myeloid progenitors in the bone marrow (Related to Fig 2). **(A)** Representative flow cytometry plots and gating strategy for granulocyte-monocyte progenitors (GMPs) in the bone marrow of naïve and infected mice at D28pi. Numbers represent the percentage in each population for one individual mouse. **(B)** Representative histograms for cell-cycle analysis on bone marrow GMPs. **(C)** Representative flow cytometry data showing Sca-1 expression on GMPs in naïve and infected mice at D28pi.
(TIF)

S3 Fig Analysis of bone marrow B cell and myeloid cells during infection (Related to Fig 3). **(A)** Representative flow cytometry plots and gating strategy for CD19^+^ B cells in the bone marrow of infected mice. Data represent mean + SEM from four mice at each time point. Similar results were obtained in a second, independent experiment. *P<0.05; **P<0.01; ***P<0.001. **(B)** Representative flow cytometry data for MHC-II and Sca-1 expression on bone marrow Ly6C^hi^ and Ly6C^lo /-^ monocytes. **(C)** Representative flow cytometry data for Galectin-3 expression on bone marrow Ly6C^hi^ monocytes.
(TIF)

S4 Fig Analysis of emergency hematopoiesis in infected Fzd6^-/-^ (KO) and Fzd6^+/+^ (WT) mice on day 21 (Related to Figs 4 and 5). Analysis of **(A-F)** bone marrow and **(G-K)** splenic HSPCs and myeloid cell subsets in infected Fzd6^-/-^ (KO) and Fzd6^+/+^ (WT) mice on day 21 post-infection. **(A)** Flow cytometry data for LSK and HSC compartments in the bone marrow. **(B)** Flow cytometry analysis of BM GMPs. Numbers in the histograms represent mean percentage. **(C)** Analysis of bone marrow myeloid subsets in the bone marrow of infected KO and WT mice. **(D)** Graph shows numbers of granulocytes and monocytes in the bone marrow. **(E)** Ly6C and CCR2 expression (MFI) on bone marrow Ly6C^hi^ monocytes. **(F)** Percentage of Sca-1^+^, MHC-II^+^ and CXCR4^+^ cells within bone marrow Ly6C^hi^ monocytes. **(G)** Flow cytometry analysis of myeloid cells in the spleen. **(H)** Numbers of myeloid cell subsets in the spleen. **(I)** Parasite burden in the spleen. **(J)** Percentage of Sca-1^+^ and MHC-II^+^ cells within Ly6C^hi^ monocytes in the spleen. **(K)** Flow cytometry data for myeloid cell subsets in the blood. Numbers indicate mean percentage of granulocytes and Ly6C^hi^ monocytes. All bar graphs represent mean + SEM with 3 mice per group for day 21pi coming from one single infection. *P<0.05; **P<0.01; ***P<0.001.
(TIF)

S5 Fig Steady-state myelopoiesis does not require Fzd6 in the bone marrow (Related to Fig 4). Analysis of bone marrow myeloid progenitor cells from naïve Fzd6^+/+^ (WT) and Fzd6^-/-^ mice (KO) mice. **(A)** BM cells were first gated on Lin^-^ (B220^-^CD3e^-^CD11b^-^GR1-Ter119^-^) and subdivided according to the expression of CD41, CD150, CD16/32 and CD105 as depicted in the representative FACS plots: CD41^+^CD150^+^, Megakaryocyte progenitors (MkP); CD16/32^hi^, CD150^-^, granulocyte-monocyte progenitors(GMP); CD105^+^CD150^+^, pre-CFU-E; CD105^+^ CD150^-^, CFU-E; CD105^-^CD150^+^, Megakaryocyte-erythrocyte progenitors (MEP); CD105^-^ CD150^-^ CD41^-^CD16/32^-^, pre-GMP (or CMP). **(B)** Total numbers for different myeloid progenitor subsets per bone marrow. **(C)** Representative flow cytometry data and mean percentages of cMOPs in naïve Fzd6^+/+^ and Fzd6^-/-^ bone marrow. **(D)** Colony forming ability of Fzd6^+/+^ and Fzd6^-/-^ bone marrow cells. Colonies were classified as erythroid (BFU-E), granulocyte (CFUG), macrophage (CFU-M), granulocyte-macrophage (CFU-GM) and granulocyte/erythroid/megakaryocyte/ macrophage (CFU-GEMM) according to size and morphology on day 12. **(E)** Flow cytometry analysis of cells recovered from CFU assays. Numbers shown in different quadrants indicate the mean percentage in CD11b^+^ cells. All histograms represent pooled data from at least three independent experiments for a total of at least five mice per group.
(TIF)

S6 Fig Analysis of myeloid progenitor cells in infected *Fzd6*^+/+^ and *Fzd6*^-/-^ bone marrow (Related to Figs 4 and 5). **(A)** Representative flow cytometry data showing GM-CSFR expression on Fzd6^+/+^ (WT) and Fzd6^-/-^ mice (KO) bone marrow LSKs and GMPs at day 28pi. Mean fluorescence intensities are depicted in the graph on the right (Mean + SEM from five mice per group). **(B)** Apoptotic rate of WT and KO HSCs determined by caspase-3 activity at D28pi. **(C-D)** Representative flow cytometry plots for Sca-1^-^ and Sca-1^+^ common monocyte progenitors (cMOPs) in naïve and infected mice.
(TIF)

S7 Fig Steady-state myeloid maturation and migration to spleen do not require Fzd6 (Related to Figs 5 and 6). Representative flow cytometry analysis of granulocytes (GR1^hi^SSC^hi^), mature monocytes (Ly6C^hi^CD11b^+^) and remaining immature/resident myelo-monocytes (Ly6Cl^o/-^ CD11b^+^) in the bone marrow **(A)** and spleens **(B)** of naive Fzd6^+/+^ (WT) and Fzd6^-/-^ mice (KO). Numbers represent the mean percentage of total bone marrow cells. Bar graphs show numbers of myeloid cell subsets in bone marrow (mean+SEM from at least three experiments for a total of at least five mice per group).
(TIF)

S8 Fig Flow cytometry analysis of bone marrow myeloid cells subsets in WT and KO mice at D28pi (Related to Figs 5 and 6). Representative flow cytometry data for **(A)** MHC-II and Sca-1; **(B)** F4-80, CCR2, CXCR4 and Arginase-1; **(C)** IL-10; and **(D)** NOS2 on Ly6C^hi^ monocytes at D28pi. Gates were determined using a combination of fluorescence-minus-one, naïve and internal negative population controls.
(TIF)

S9 Fig Liver monocytes are also decreased in number in Leishmania-infected Fzd6^-/-^ mice (Related to Fig 6). Analysis of myeloid cell subsets in the livers of infected Fzd6^-/-^ (KO) and Fzd6^+/+^ (WT) mice on day 28pi. **(A)** Representative flow cytometry data shows granulocytes, monocytes and macrophages in the liver. Mean percentage for each cell subset is indicated within flow cytometry plots. **(B)** Graph show numbers of granulocytes and monocytes. **(C)** Percentage of F4-80^+^ cells within Ly6C^hi^ monocytes and numbers of Ly6C^lo/-^ F4-80^+^ macrophages in the liver. **(D)** Parasite burden expressed as LDU in the liver on day 28pi. **(E)** Ly6C and CCR2 expression (MFI) on Ly6C^hi^ monocytes in the liver. **(F)** Percentage of Sca-1^+^ and MHC-II^+^ cells within Ly6C^hi^ monocytes in the liver. All bar graphs represent mean + SEM with 7 mice per group for day 28pi coming from one single infection. Similar results were obtained in a second, independent experiment. *P<0.05; **P<0.01; ***P<0.001.
(TIF)

S10 Fig Analysis of T lymphocytes and bone marrow-derived macrophages from *Fzd6*^-/-^ and *Fzd6*^+/+^ mice (Related to Fig 7). **(A, C)** Representative flow cytometry data for lymphoid cell subsets in **(A)** bone marrow and **(C)** spleen of naïve and infected *Fzd6*^-/-^ (KO) and *Fzd6*^+/+^ (WT) mice. Numbers in flow cytometry plots indicate mean percentage for CD19^+^ B cells, total CD3ε^+^ T cells and CD3ε^+^ CD4^+^ and CD3ε^+^CD8^+^ T cells within BM. **(B, D)** Numbers of CD19^+^ B cells and CD3ε^+^ T cells in **(B)** BM and **(D)** spleen of naïve and infected mice on day 28. **(E)** Representative flow cytometry histograms showing uniform CD11b and F4/80 expression on untreated and infected macrophages. **(F)** MHC-II and Sca-1 expression on untreated, INF-γ stimulated and infected macrophages. **(G)** Flow cytometry analysis of parasite uptake at 24h. Similar results were obtained from three independent experiments.
(TIF)

S11 Fig Extracellular fluid from naive bone marrow does not promote myeloid differentiation (Related to Fig 9). Impact of bone marrow supernatants from naïve mice as compared to *L*. *donovani*–infected mice after four days of culture on **(A)** Lin^-^ *Fzd6*^+/+^ (WT) BM cells and **(B)** Lin^-^ *Fzd6*^-/-^ (KO) BM cells. **(C)** Freshly isolated lineage-depleted *Fzd6*^-/-^ (KO) BM cells were cultured in complete medium supplemented with 30% BM supernatant as indicated. Representative flow cytometry data show the gating strategy for CD11b^+^, LSK and GMP populations. Graphs show numbers of cell recovered per 5x10^5^ cells seeded for each subset.
(TIF)

S1 Table Antibodies used in flow cytometry. (PDF)
