# Supplementary figures and images for: Infection-adapted emergency hematopoiesis promotes visceral leishmaniasis
Source: PLoS Pathog. 2017 Aug 7;13(8):e1006422. doi: 10.1371/journal.ppat.1006422 (PMC5560750; doi:10.1371/journal.ppat.1006422)

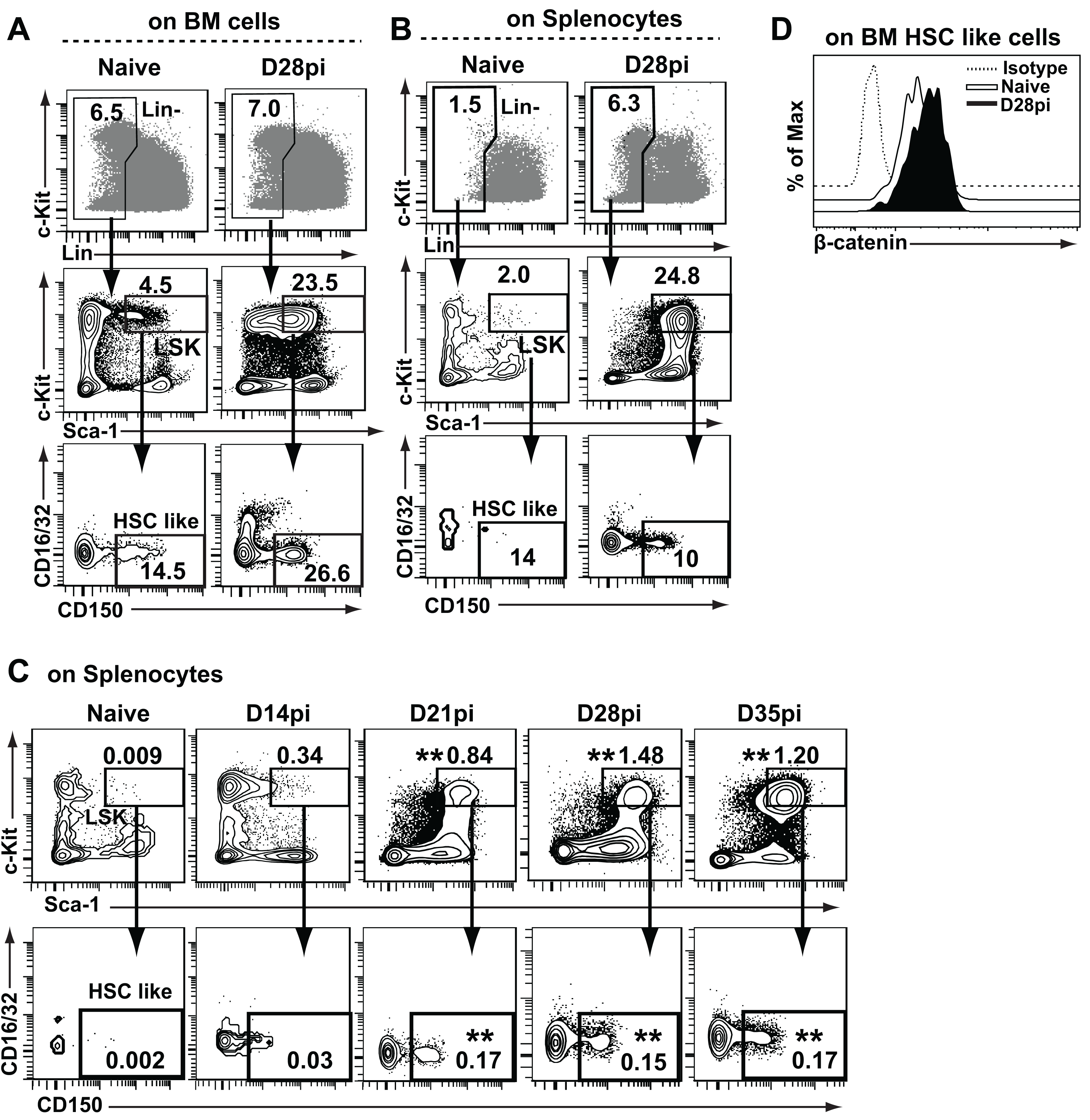

Supplement: S1 Fig — (A-B) Representative flow cytometry plots and gating strategy for LSKs and HSCs in the bone marrow and spleen. Numbers represent the percentage in each population for one individual mouse on day 28. (C) Representative flow cytometry plots and gating strategy for LSKs and HSCs in spleens of infected mice at various time points during infection. Mean percentage for each cell subset is indicated in histograms. (D) Intracellular β-catenin expression on bone marrow HSC cell like subsets in naïve and infected mice at D28pi. (TIF) [file ppat.1006422.s001.tif]

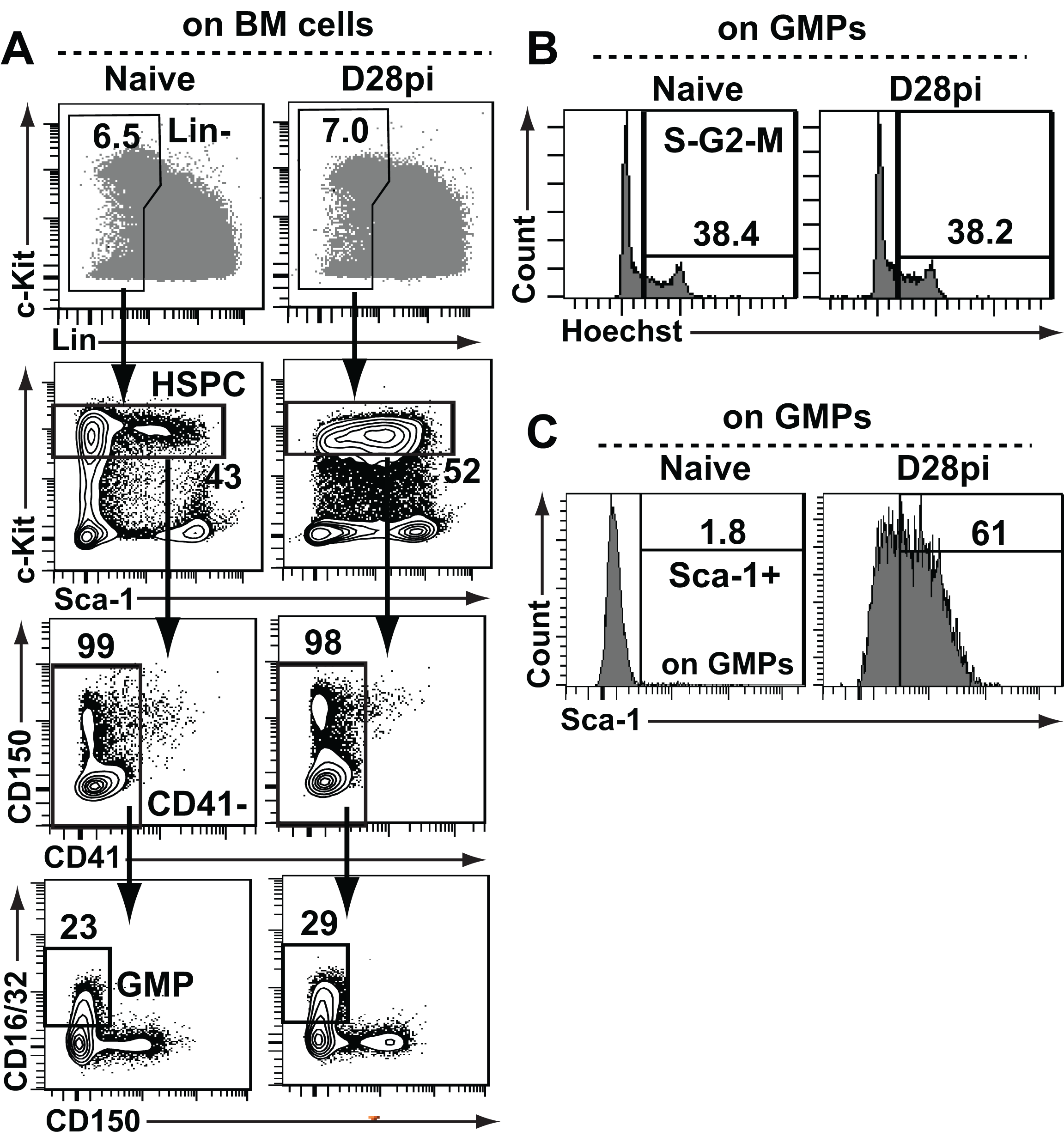

Supplement: S2 Fig — (A) Representative flow cytometry plots and gating strategy for granulocyte-monocyte progenitors (GMPs) in the bone marrow of naïve and infected mice at D28pi. Numbers represent the percentage in each population for one individual mouse. (B) Representative histograms for cell-cycle analysis on bone marrow GMPs. (C) Representative flow cytometry data showing Sca-1 expression on GMPs in naïve and infected mice at D28pi. (TIF) [file ppat.1006422.s002.tif]

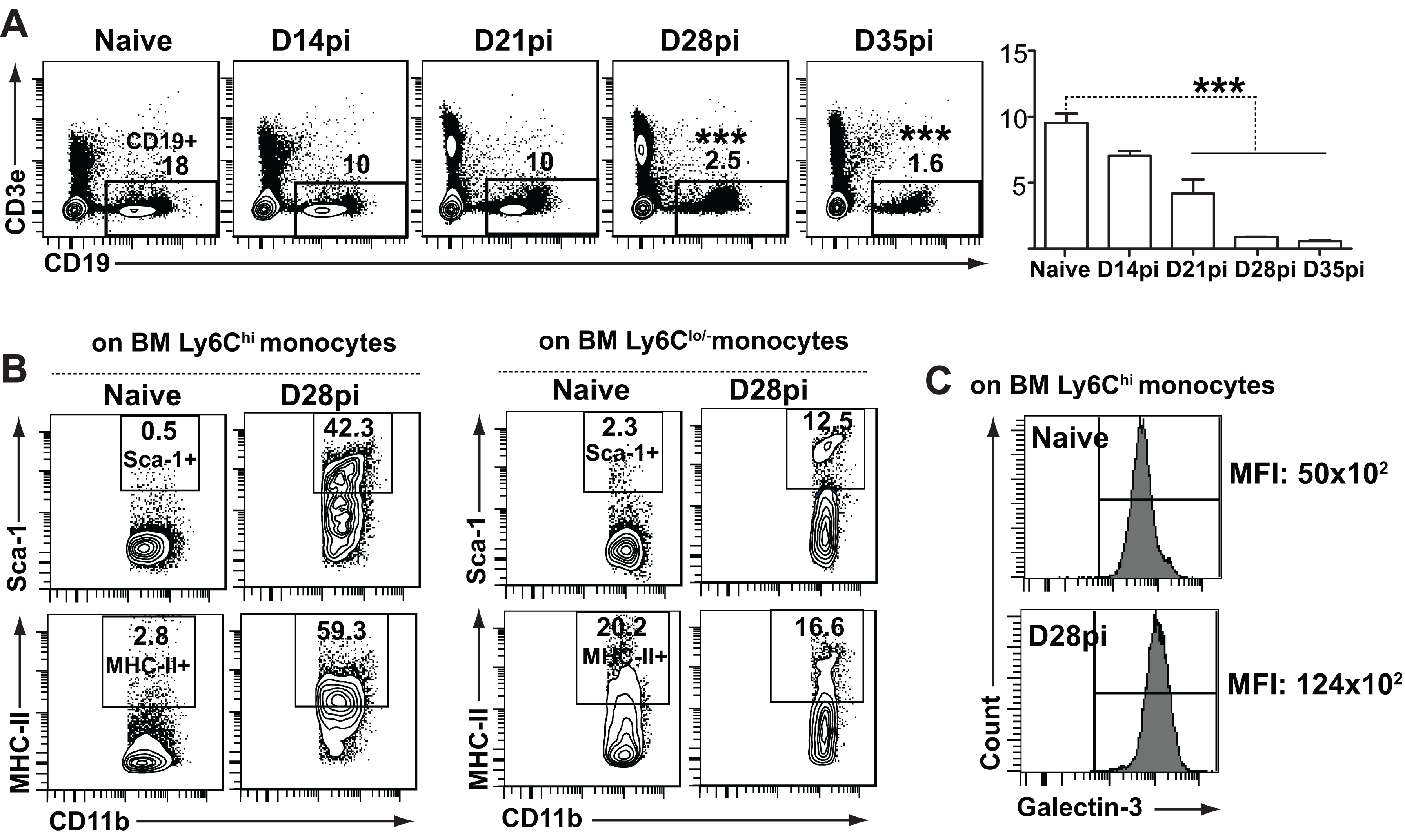

Supplement: S3 Fig — (A) Representative flow cytometry plots and gating strategy for CD19+ B cells in the bone marrow of infected mice. Data represent mean + SEM from four mice at each time point. Similar results were obtained in a second, independent experiment. *P<0.05; **P<0.01; ***P<0.001. (B) Representative flow cytometry data for MHC-II and Sca-1 expression on bone marrow Ly6Chi and Ly6Clo /- monocytes. (C) Representative flow cytometry data for Galectin-3 expression on bone marrow Ly6Chi monocytes. (TIF) [file ppat.1006422.s003.tif]

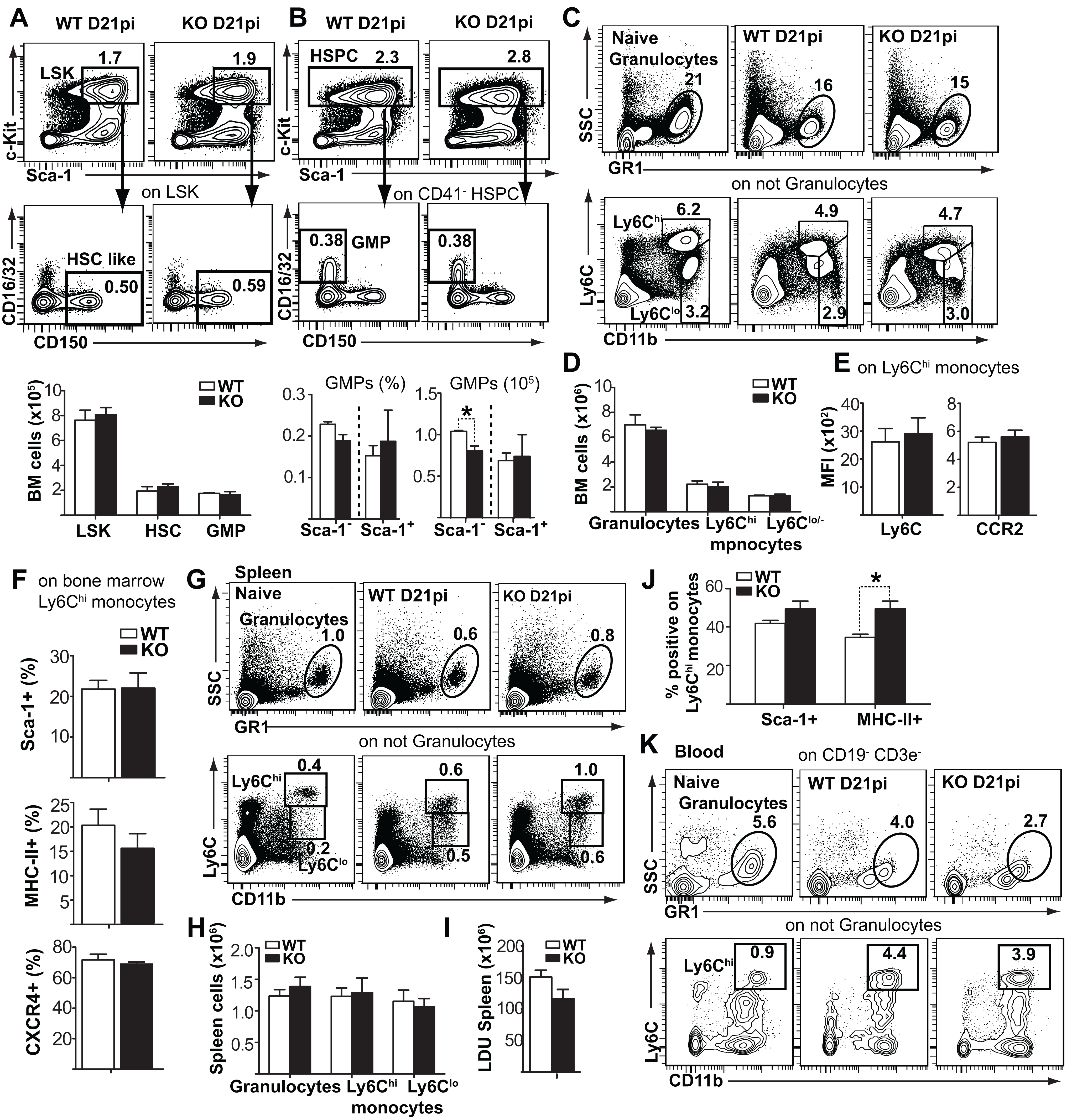

Supplement: S4 Fig — Analysis of (A-F) bone marrow and (G-K) splenic HSPCs and myeloid cell subsets in infected Fzd6-/- (KO) and Fzd6+/+ (WT) mice on day 21 post-infection. (A) Flow cytometry data for LSK and HSC compartments in the bone marrow. (B) Flow cytometry analysis of BM GMPs. Numbers in the histograms represent mean percentage. (C) Analysis of bone marrow myeloid subsets in the bone marrow of infected KO and WT mice. (D) Graph shows numbers of granulocytes and monocytes in the bone marrow. (E) Ly6C and CCR2 expression (MFI) on bone marrow Ly6Chi monocytes. (F) Percentage of Sca-1+, MHC-II+ and CXCR4+ cells within bone marrow Ly6Chi monocytes. (G) Flow cytometry analysis of myeloid cells in the spleen. (H) Numbers of myeloid cell subsets in the spleen. (I) Parasite burden in the spleen. (J) Percentage of Sca-1+ and MHC-II+ cells within Ly6Chi monocytes in the spleen. (K) Flow cytometry data for myeloid cell subsets in the blood. Numbers indicate mean percentage of granulocytes and Ly6Chi monocytes. All bar graphs represent mean + SEM with 3 mice per group for day 21pi coming from one single infection. *P<0.05; **P<0.01; ***P<0.001. (TIF) [file ppat.1006422.s004.tif]

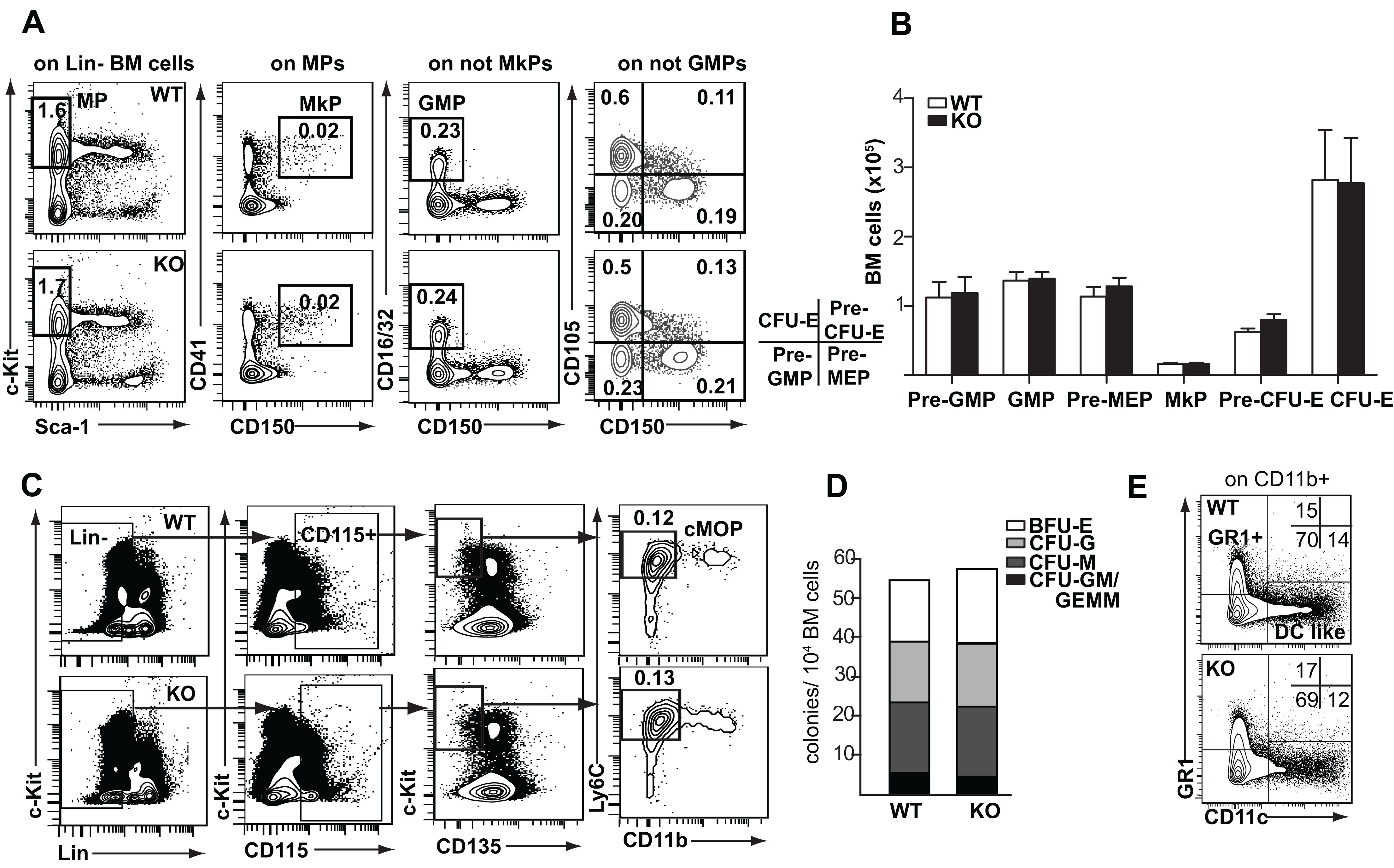

Supplement: S5 Fig — Analysis of bone marrow myeloid progenitor cells from naïve Fzd6+/+ (WT) and Fzd6-/- mice (KO) mice. (A) BM cells were first gated on Lin- (B220-CD3e-CD11b-GR1-Ter119-) and subdivided according to the expression of CD41, CD150, CD16/32 and CD105 as depicted in the representative FACS plots: CD41+CD150+, Megakaryocyte progenitors (MkP); CD16/32hi, CD150-, granulocyte-monocyte progenitors(GMP); CD105+CD150+, pre-CFU-E; CD105+ CD150-, CFU-E; CD105-CD150+, Megakaryocyte-erythrocyte progenitors (MEP); CD105- CD150- CD41-CD16/32-, pre-GMP (or CMP). (B) Total numbers for different myeloid progenitor subsets per bone marrow. (C) Representative flow cytometry data and mean percentages of cMOPs in naïve Fzd6+/+ and Fzd6-/- bone marrow. (D) Colony forming ability of Fzd6+/+ and Fzd6-/- bone marrow cells. Colonies were classified as erythroid (BFU-E), granulocyte (CFUG), macrophage (CFU-M), granulocyte-macrophage (CFU-GM) and granulocyte/erythroid/megakaryocyte/ macrophage (CFU-GEMM) according to size and morphology on day 12. (E) Flow cytometry analysis of cells recovered from CFU assays. Numbers shown in different quadrants indicate the mean percentage in CD11b+ cells. All histograms represent pooled data from at least three independent experiments for a total of at least five mice per group. (TIF) [file ppat.1006422.s005.tif]

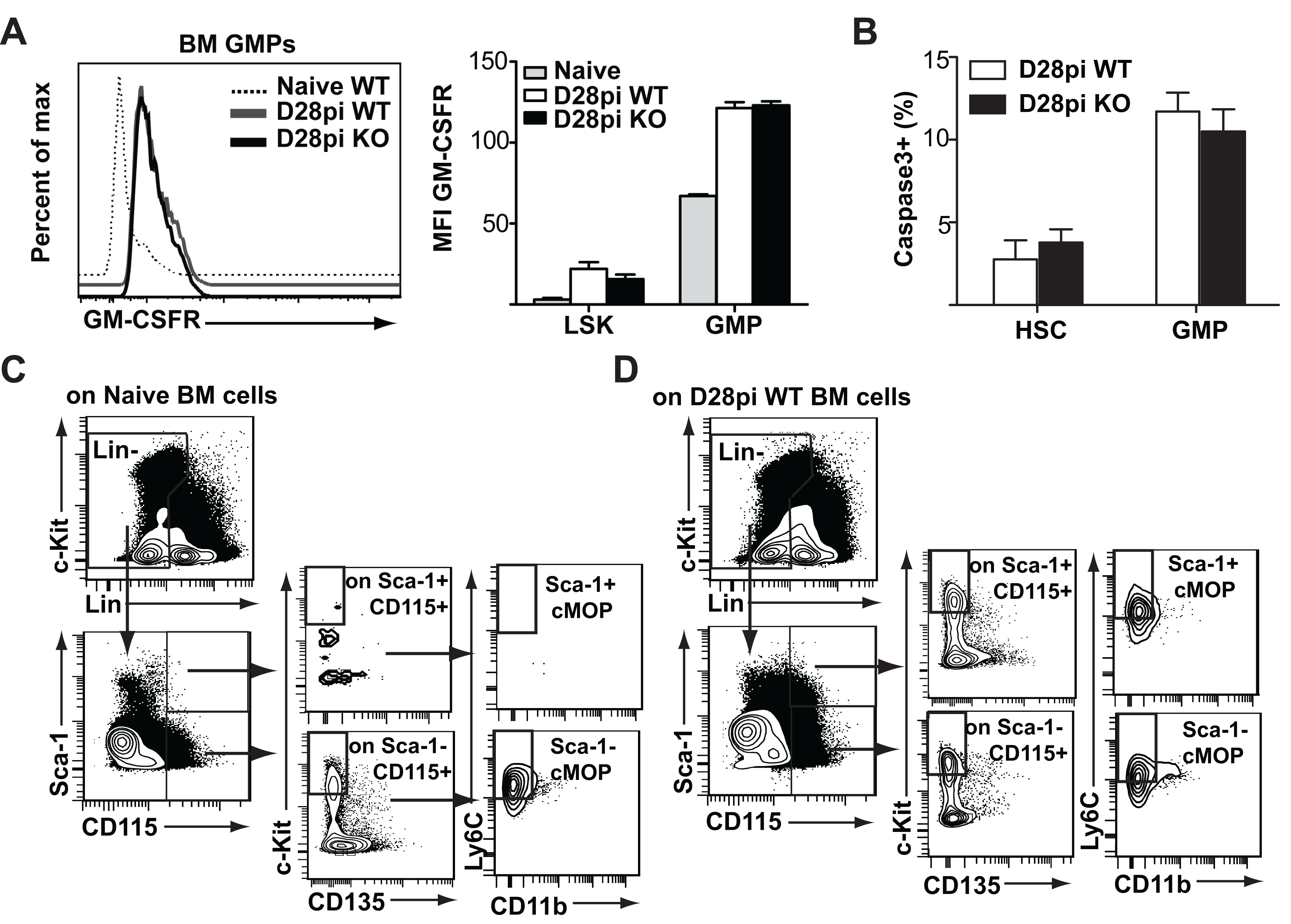

Supplement: S6 Fig — (A) Representative flow cytometry data showing GM-CSFR expression on Fzd6+/+ (WT) and Fzd6-/- mice (KO) bone marrow LSKs and GMPs at day 28pi. Mean fluorescence intensities are depicted in the graph on the right (Mean + SEM from five mice per group). (B) Apoptotic rate of WT and KO HSCs determined by caspase-3 activity at D28pi. (C-D) Representative flow cytometry plots for Sca-1- and Sca-1+ common monocyte progenitors (cMOPs) in naïve and infected mice. (TIF) [file ppat.1006422.s006.tif]

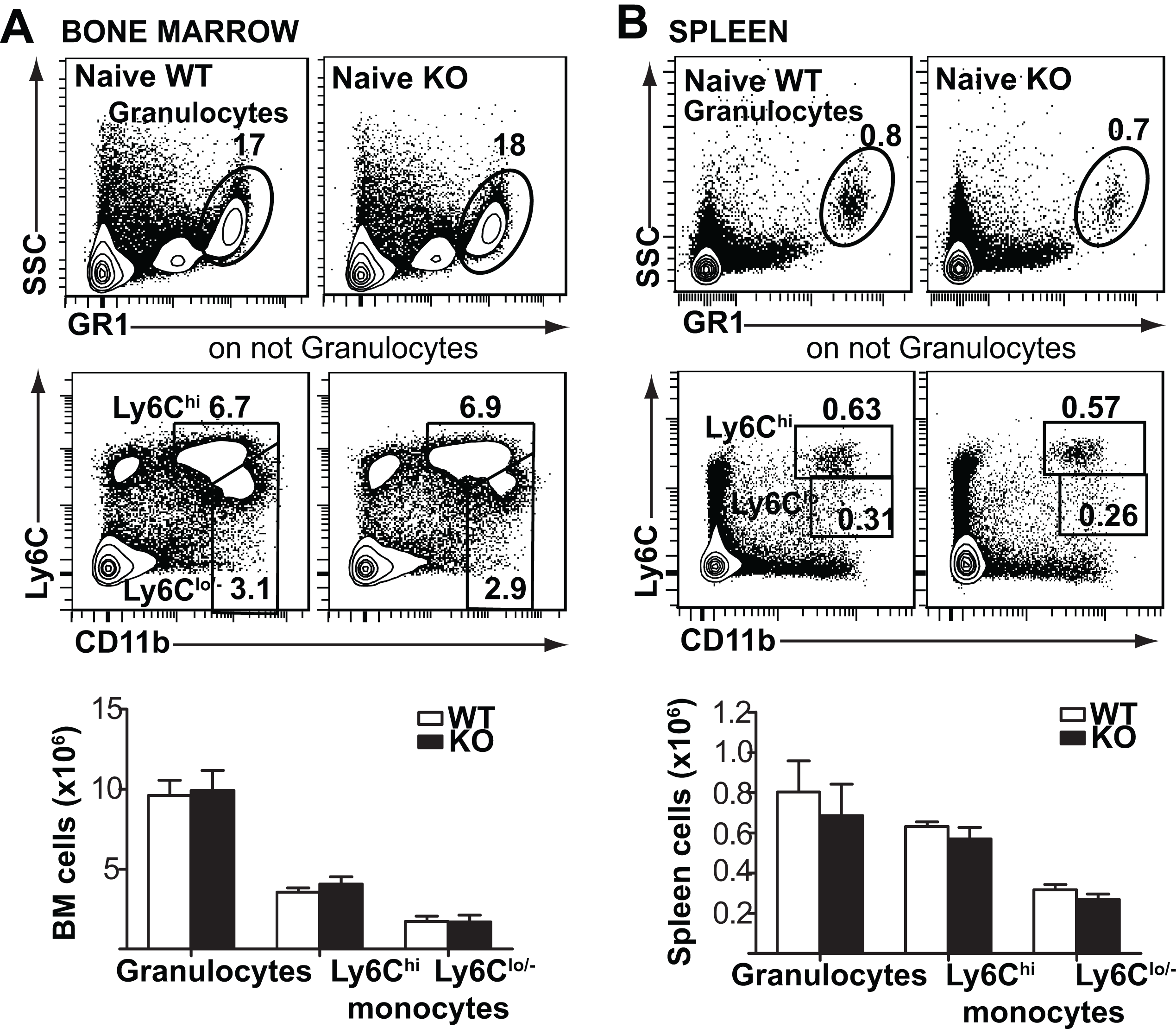

Supplement: S7 Fig — Representative flow cytometry analysis of granulocytes (GR1hiSSChi), mature monocytes (Ly6ChiCD11b+) and remaining immature/resident myelo-monocytes (Ly6Clo/- CD11b+) in the bone marrow (A) and spleens (B) of naive Fzd6+/+ (WT) and Fzd6-/- mice (KO). Numbers represent the mean percentage of total bone marrow cells. Bar graphs show numbers of myeloid cell subsets in bone marrow (mean+SEM from at least three experiments for a total of at least five mice per group). (TIF) [file ppat.1006422.s007.tif]

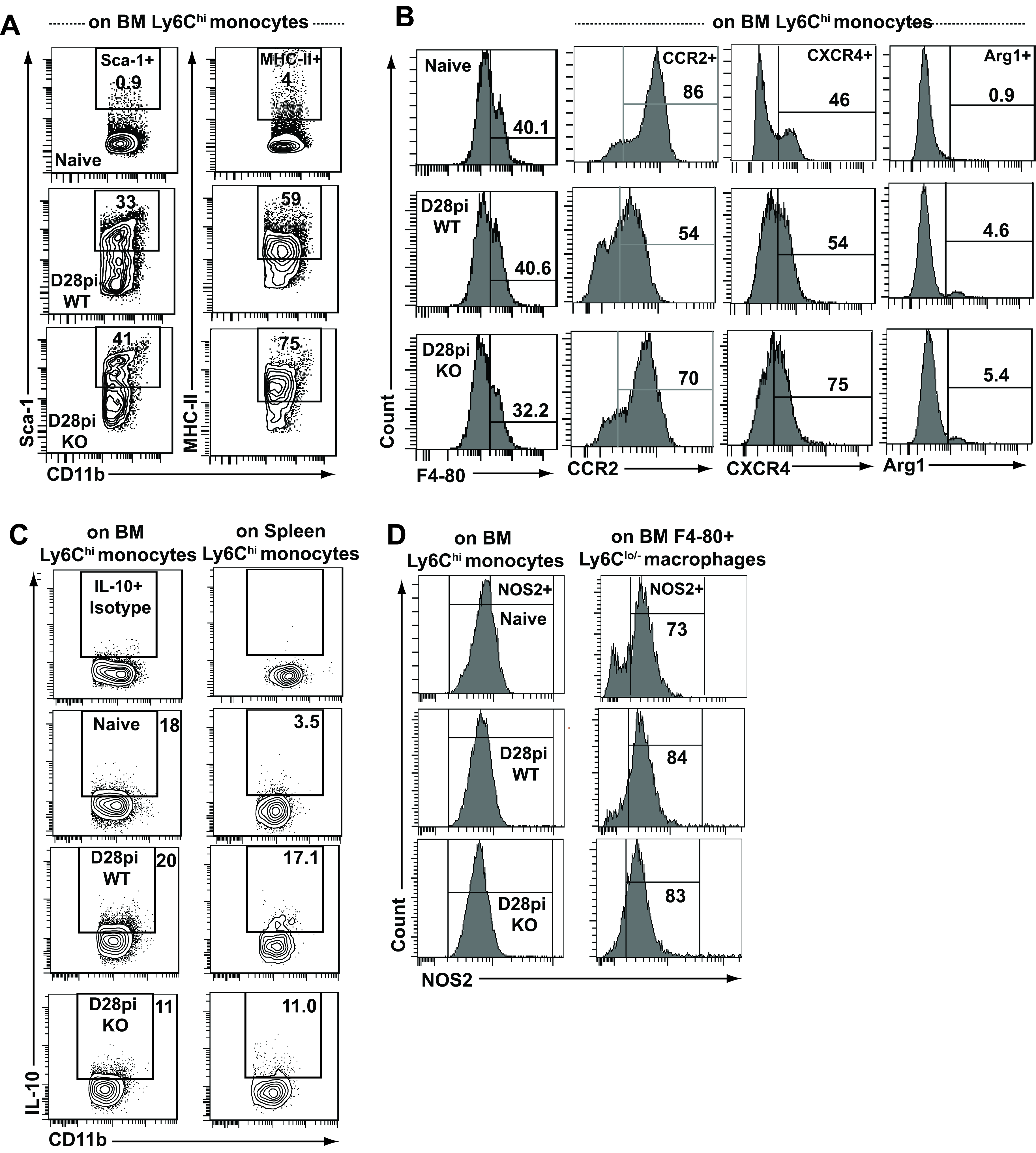

Supplement: S8 Fig — Representative flow cytometry data for (A) MHC-II and Sca-1; (B) F4-80, CCR2, CXCR4 and Arginase-1; (C) IL-10; and (D) NOS2 on Ly6Chi monocytes at D28pi. Gates were determined using a combination of fluorescence-minus-one, naïve and internal negative population controls. (TIF) [file ppat.1006422.s008.tif]

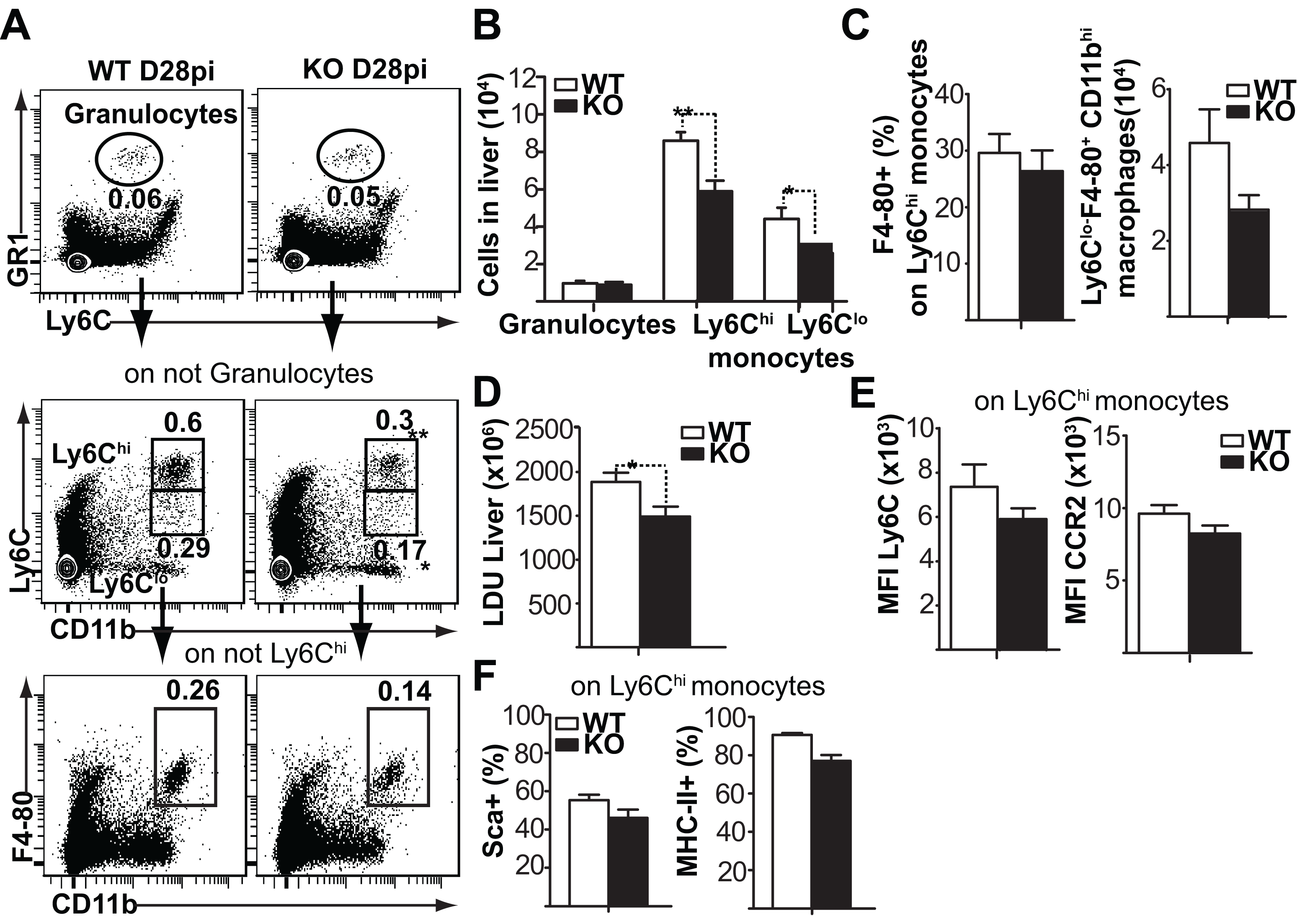

Supplement: S9 Fig — Analysis of myeloid cell subsets in the livers of infected Fzd6-/- (KO) and Fzd6+/+ (WT) mice on day 28pi. (A) Representative flow cytometry data shows granulocytes, monocytes and macrophages in the liver. Mean percentage for each cell subset is indicated within flow cytometry plots. (B) Graph show numbers of granulocytes and monocytes. (C) Percentage of F4-80+ cells within Ly6Chi monocytes and numbers of Ly6Clo/- F4-80+ macrophages in the liver. (D) Parasite burden expressed as LDU in the liver on day 28pi. (E) Ly6C and CCR2 expression (MFI) on Ly6Chi monocytes in the liver. (F) Percentage of Sca-1+ and MHC-II+ cells within Ly6Chi monocytes in the liver. All bar graphs represent mean + SEM with 7 mice per group for day 28pi coming from one single infection. Similar results were obtained in a second, independent experiment. *P<0.05; **P<0.01; ***P<0.001. (TIF) [file ppat.1006422.s009.tif]

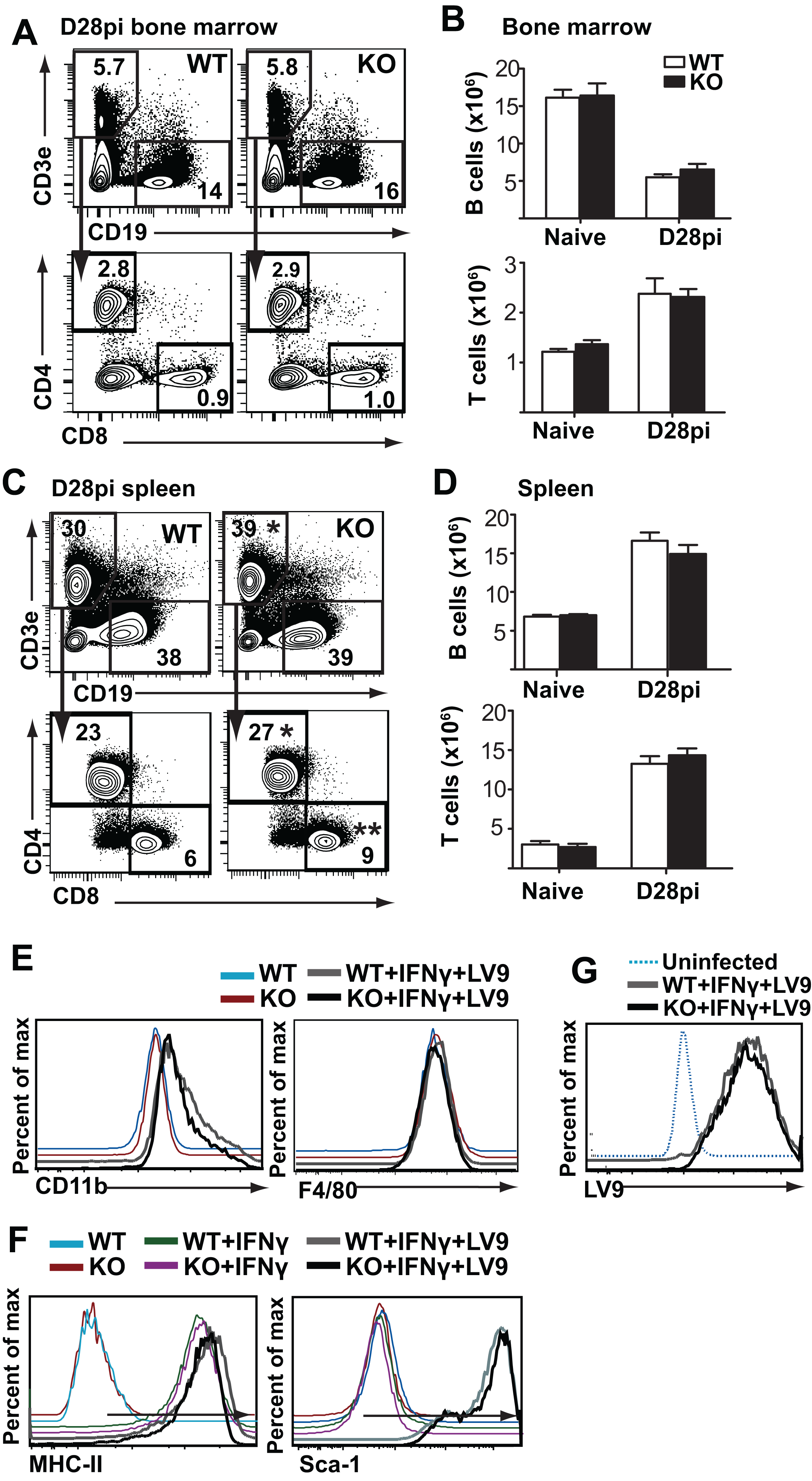

Supplement: S10 Fig — (A, C) Representative flow cytometry data for lymphoid cell subsets in (A) bone marrow and (C) spleen of naïve and infected Fzd6-/- (KO) and Fzd6+/+ (WT) mice. Numbers in flow cytometry plots indicate mean percentage for CD19+ B cells, total CD3ε+ T cells and CD3ε+ CD4+ and CD3ε+CD8+ T cells within BM. (B, D) Numbers of CD19+ B cells and CD3ε+ T cells in (B) BM and (D) spleen of naïve and infected mice on day 28. (E) Representative flow cytometry histograms showing uniform CD11b and F4/80 expression on untreated and infected macrophages. (F) MHC-II and Sca-1 expression on untreated, INF-γ stimulated and infected macrophages. (G) Flow cytometry analysis of parasite uptake at 24h. Similar results were obtained from three independent experiments. (TIF) [file ppat.1006422.s010.tif]

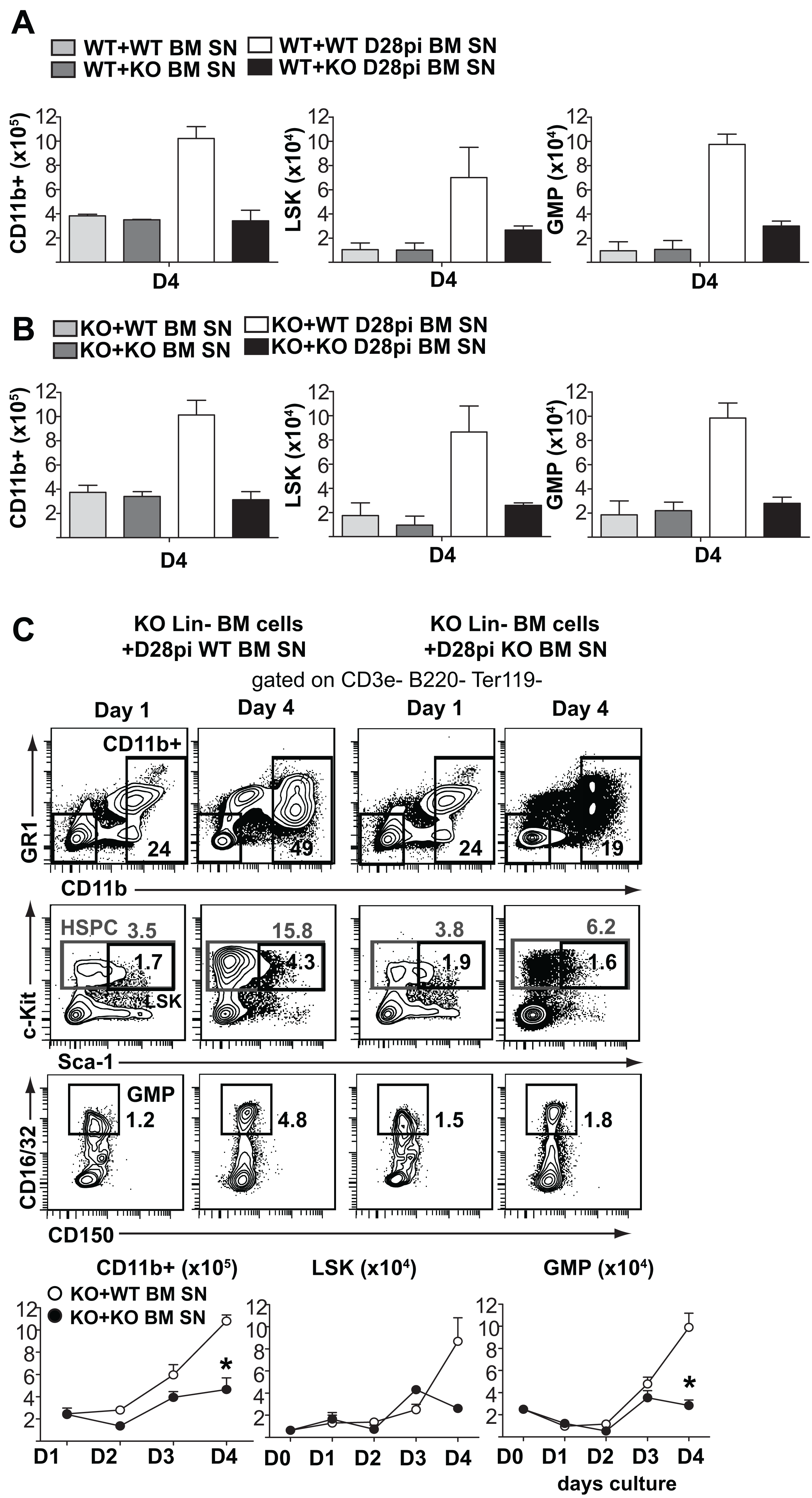

Supplement: S11 Fig — Impact of bone marrow supernatants from naïve mice as compared to L. donovani–infected mice after four days of culture on (A) Lin- Fzd6+/+ (WT) BM cells and (B) Lin- Fzd6-/- (KO) BM cells. (C) Freshly isolated lineage-depleted Fzd6-/- (KO) BM cells were cultured in complete medium supplemented with 30% BM supernatant as indicated. Representative flow cytometry data show the gating strategy for CD11b+, LSK and GMP populations. Graphs show numbers of cell recovered per 5x10^5^ cells seeded for each subset. (TIF) [file ppat.1006422.s011.tif]
