# Supplementary material for: Infection-adapted emergency hematopoiesis promotes visceral leishmaniasis
Source: PLoS Pathog. 2017 Aug 7;13(8):e1006422. doi: 10.1371/journal.ppat.1006422 (PMC5560750; doi:10.1371/journal.ppat.1006422)
Supplement: S1 Table — (PDF) [file ppat.1006422.s012.pdf]

Antigen	Fluorochrome	Clone	Supplier
**Active caspase-3**	Alexa Fluor 488	-	Cell Signaling Technologies
**Active β-catenin**	Unconjugated	D13A1	Cell Signaling Technologies
**Arginase-1**	PE	-	R&D Systems, Minneapolis, MN
**CCR2**	Alexa Fluor 700	-	R&D Systems
**CD105**	Pacific Blue	MJ7-I8	eBioscience, San Diego, CA
**CD115**	PE-Cy7, FITC	AFS98	eBioscience
**CD117 (cKit)**	PE	2B8	BD Biosciences, Mississauga, ON, Canada
**CD11b**	Biotin	M1/70	BD Biosciences
**CD11b**	Alexa Fluor 647	M1/70	eBioscience
**CD135 (Flt3)**	PerCP–eFluor 710	A2F10	BD Biosciences
**CD150**	Alexa Fluor 647	TC15-12F12.2	BD Biosciences; BioLegend, San Diego, CA
**CD16/32**	PerCP-Cy5.5	93	eBioscience
**CD19**	PE	1D3	eBioscience
**CD3e**	Biotin	145-2C11	BD Biosciences
**CD3e**	PE-Cy7	145-2C11	eBioscience
**CD4**	APC-eFluor 450	RM4-5	eBioscience
**CD41**	PE, FITC	MWReg30	BD Biosciences
**CD45/B220**	Biotin	RA3-6B2	BD Biosciences
**CD48**	PerCP-Cy5.5	HM48-1	BioLegend
**CD8**	eFluor 450	53-6.7	eBioscience
**CXCR4**	PE	2B11	BD Biosciences
**F4/80**	PE-Cy7	BM8	BioLegend
**Galectin3**	PE	eBioM3/38	eBioscience
**GM-CSFR Rα**	Alexa Fluor 700	-	R&D Systems
**GR1**	Biotin	RB6-8C5	BD Biosciences
**GR1**	APC-eFluor 780	RB6-8C5	eBioscience
**Hoechst**		33342	Sigma-Aldrich, Oakville, ON, Canada
**IFN-γ**	APC	-	BD Biosciences
**IL-10**	PE	-	BD Biosciences
**iNOS**	APC	CXNFT	eBioscience
**Ki67**	PE–eFluor 610	SolA15	eBioscience
**Ly6C**	PerCP-Cy5.5, FITC	AL-21	eBioscience; BD Biosciences
**Ly6G**	Biotin	A8	BioLegend
**MHC-II**	PE	MS/114.15.2	eBioscience
**NK-1.1**	Biotin	PK136	BD Biosciences
**Sca-1 (Ly6A/E)**	PE-Cy7, BV711	D7	BD Biosciences
**Streptavidin**	BD Horizon V500, FITC	-	BD Biosciences
**Ter119**	Biotin	-	BD Biosciences
**TNF-α**	PE-Cy7	MP6-XT22	BD Biosciences

Table S1. Antibodies used in flow cytometry (Related to Materials and Methods)
